# Reductions of aldehydes and ketones with a readily available N-heterocyclic carbene borane and acetic acid

**DOI:** 10.3762/bjoc.9.76

**Published:** 2013-04-08

**Authors:** Vladimir Lamm, Xiangcheng Pan, Tsuyoshi Taniguchi, Dennis P Curran

**Affiliations:** 1 Department of Chemistry, University of Pittsburgh, Pittsburgh, PA 15260 USA

**Keywords:** NHC-borane, N-heterocyclic carbene borane, reduction

## Abstract

Acetic acid promotes the reduction of aldehydes and ketones by the readily available N-heterocyclic carbene borane, 1,3-dimethylimidazol-2-ylidene borane. Aldehydes are reduced over 1–24 h at room temperature with 1 equiv of acetic acid and 0.5 equiv of the NHC-borane. Ketone reductions are slower but can be accelerated by using 5 equiv of acetic acid. Aldehydes can be selectively reduced in the presence of ketones. On a small scale, products are isolated by evaporation of the reaction mixture and direct chromatography.

## Introduction

Reduction of carbonyl compounds is a common, fundamental chemical transformation. Among the numerous hydride reagents available for such reductions, boron reductants are widely used in the field of synthetic chemistry due to their availability and favorable reaction profiles [1–3]. For instance, sodium borohydride (NaBH_4_) is an inexpensive salt that is one of the most popular hydride sources for the reduction of aldehydes and ketones [4]. Reactions with NaBH_4_ are usually performed in a protic solvent such as methanol, and quenching and aqueous workup are essential steps along the way to isolation of the product. Although many borohydride reagents are relatively stable solids, their contact with moisture can cause decomposition with the release of hydrogen gas. Therefore, appropriate precautions are required for larger scale reactions as well as during transport and storage [5].

N-Heterocyclic carbene boranes (NHC-boranes) have emerged in recent years as a useful class of synthetic reagents, which have interesting chemistry in their own right [6–7]. Most NHC complexes of borane (NHC-BH_3_) are white solids that are convenient to handle because they are stable to air, water, strong bases, and weak acids [8]. Unlike amine-boranes such as R_3_N-BH_3_ or pyridine-BH_3_ [9–11], carbene-boranes (NHC-BH_3_) do not decomplex easily to release reactive borane (BH_3_) to solution under typical thermal reaction conditions. Perhaps because of their stability, the potential use of carbene-boranes as hydride sources has been largely overlooked. Lindsay and McArthur reported that strong Lewis acids such as BF_3_ and Sc(OTf)_3_ activate ketones towards reduction by achiral and chiral carbene boranes [12], and we showed that some high-temperature reductions of halides were probably occurring by ionic rather than radical pathways [13].

**Scheme 1:**
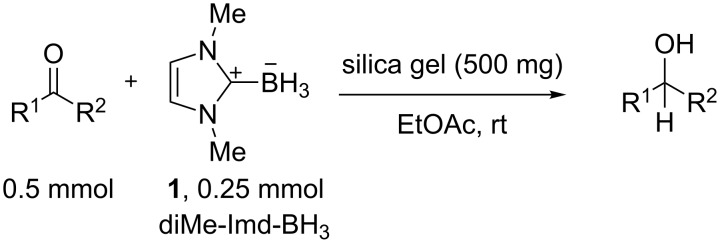
Aldehydes and ketones are reduced by **1** when silica gel is added.

Recently, we discovered in collaboration with Mayr that NHC-boranes are good hydride sources by measurement of the nucleophilicity parameter, *N* [14]. By this measure, NHC-boranes are among the best neutral hydride donors known, more reactive than distant relatives such as silanes, stannanes, and dihydropyridines, and more reactive even than their amine-borane cousins [15–19]. Despite being neutral, 1,3-dimethylimidazol-2-ylidene borane (diMe-Imd-BH_3_, **1**, see Scheme 1) has an *N* value (*N* = 12) that is roughly comparable to the anion cyanoborohydride (NaBH_3_CN) in DMSO. Unlike NaBH_3_CN, diMe-Imd-BH_3_ (**1**) is freely soluble in many organic solvents, including most aprotic solvents. Unaided reductions of aldehydes and ketones by **1** are not practical (little or no reaction occurs, depending on the substrate). However, addition of silica gel causes reductions to occur at ambient temperatures (Scheme 1) [20]. In a preferred small-scale procedure, 0.5 mmol of an aldehyde or ketone can be reduced by 0.25 mmol of **1** and 500 mg of added silica gel over periods ranging from 1–24 h.

## Results and Discussion

**Scheme 2:**
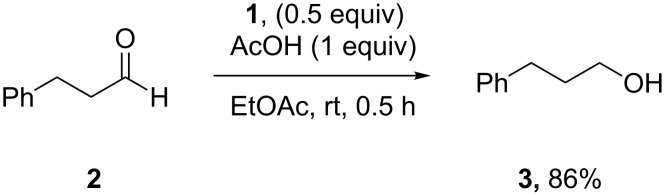
Pilot reduction of aldehyde **2** with **1** and acetic acid.

DiMe-Imd-BH_3_ (**1**) does not react with silica gel, so it seems likely that silica gel serves as a weak acid to activate carbonyl groups toward reduction by **1**. Based on this analysis, we decided to replace the silica gel with weak acids in order to extend the practicality of reductions with NHC-boranes. We specifically targeted acetic acid because it is a weak acid that is cheap, soluble and volatile. A pilot reduction of 3-phenylpropanal **2** (0.5 mmol), **1** (0.5 equiv), and acetic acid (1.0 equiv) in ethyl acetate (EtOAc) was complete in 30 min and gave 3-phenylpropanol (**3**) in 86% yield after evaporation and flash chromatography (Scheme 2). Other acids including pyridinium *p*-toluenesulfonate, benzohydroxamic acid, and 2,2-dimethyl-1,3-dioxane-4,6-dione (Meldrum’s acid) also promoted this reduction, but offered no clear advantages over acetic acid. No reduction occurs over 24 h when **1** and **2** are stirred in EtOAc without acid under these conditions.

**Figure 1:**
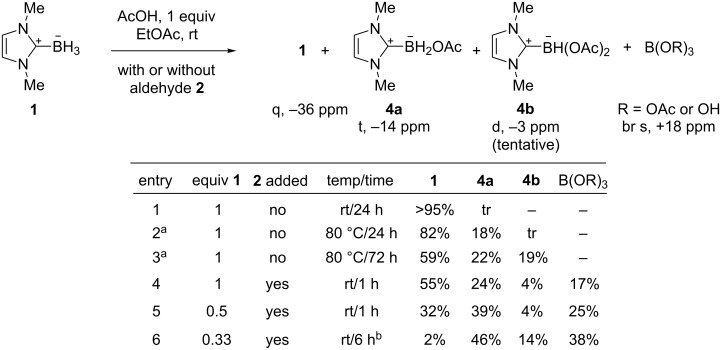
^11^B NMR studies of boron products from **1** and acetic acid with and without aldehyde **2**. ^a^In C_6_D_6_; ^b^unreacted aldehyde remained.

We next conducted a series of experiments to look at the boron products of such reactions by ^11^B NMR spectroscopy, and these results are summarized in Figure 1. A control experiment showed that the direct acid/base reaction of **1** with acetic acid (aldehyde omitted) was very slow at rt (entry 1). Evolution of hydrogen gas was not evident (no bubbling), and boryl acetate **4a** was present in only trace amounts (<<5%) after 24 h. Evidently then, the acetic acid functions as a Brønsted acid to activate the carbonyl group towards attachment by **1**. This is different from NaBH_4_, which reacts quickly with acetic acid to liberate H_2_ and form a modified reducing agent, NaBH(OAc)_3_ [21–22].

Even with heating, the direct acid/base reaction of **2** and acetic acid was rather slow [23]. After one day (entry 2), about 18% of **2** was converted to the known boryl acetate **4a** (−14 ppm) [14]. After three days (entry 3), the major component was still the starting NHC-borane **2**, but now there was 22% of **4a** and 19% of a new product that exhibited a broad doublet at −3 ppm. We could not isolate this product by flash chromatography, but we tentatively assign it as boryl diacetate **4b**. For comparison, the known ditriflate dipp-Imd-BH(OTf)_2_ resonates at −2 ppm [24].

Next we reduced hydrocinnamaldehyde **2** (1 equiv) under the standard procedure (AcOH, 1 equiv) but varying the amount of NHC-borane **1**. Alcohol **3** is the reduction product, and the soluble boron products [25] were identified by ^11^B NMR spectroscopy of the crude product mixture. Each product spectrum gave the same four resonances, but in different ratios. With 1 equiv of **1** (entry 4), there was 55% of the starting NHC-borane **1**, 24% boryl monoacetate **4a**, 4% boryl diacetate **4b**, and 17% of a broad resonance at about +18 ppm. This is not in the region expected for an NHC-borane, but is instead where boric acid and similar molecules resonance. Thus, it might be B(OAc)_3_, but it could also be boric acid itself (the solvent was not dried) or some intermediate product. We write this simply as B(OR)_3_. With 0.5 equiv **1** (entry 5), there was 32% of the starting **1**, 39% **4a**, 4% **4b** and 25% B(OR)_3_. The final reaction with 0.33 equiv of **1** was considerably slower (entry 6). Aldehyde **2** still remained after 6 h, and the ^11^B NMR spectrum shows 2% starting **1**, 46% **4a**, 14% **4b** and 38% B(OR)_3_. These results demonstrate that the reaction is complex on the boron side, with the second and third hydride transfers competing with the first. This makes little difference when the alcohol is the target product because all the other boron and NHC products are held back on the silica gel during flash chromatography.

To compare the acid-promoted reductions with the silica-gel-promoted reactions, we tested the scope of this reaction on many of the same aldehydes and ketones (Table 1 and Table 2). The typical procedure is convenient, and no precautions were taken to exclude moisture or air. For aldehyde reductions (Table 1), the reactants were mixed in EtOAc that was taken from the bottle without drying. The standard ratio of aldehyde to **1** was 1:0.5, so two of the three hydride equivalents of **1** are consumed. After completion of the reaction, the solvent was removed on a rotary evaporator, and the residue was directly purified by silica gel chromatography.

**Table 1:** Reductions of aldehydes by diMe-Imd-BH_3_ (**1**) and acetic acid.^a^

entry	aldehyde	alcohol	yield

1	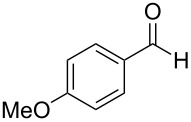 **5**	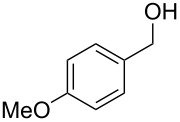 **12**	92%
2	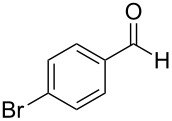 **6**	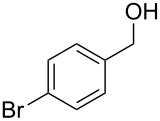 **13**	79%
3	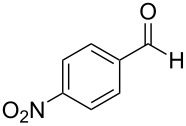 **7**	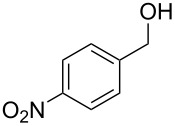 **14**	89%^b^
4	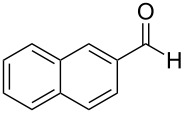 **8**	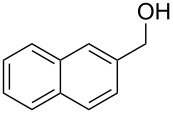 **15**	93%
5	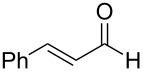 **9**	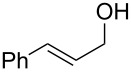 **16**	86%
6	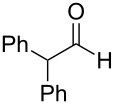 **10**	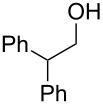 **17**	85%
7	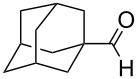 **11**	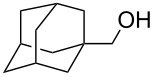 **18**	84%^b^

^a^Conditions: aldehyde (0.5 mmol), **1** (0.25 mmol), and AcOH (0.5 mmol) in EtOAc (2 mL) at room temperature for 24 h; ^b^reaction time was 1 h.

Aromatic aldehydes **5**–**8** gave the corresponding benzylic alcohols **12**–**15** in yields of 79–93% (Table 1, entries 1–4). Cinnamaldehyde underwent exclusive 1,2-reduction to give allyl alcohol **16** in 86% (entry 5). Branched aldehydes **10** and **11** were also reduced to the corresponding primary alcohols **17** and **18** in 85% and 84% yield, respectively (entries 6 and 7).

To be consistent with the prior silica gel reductions, ketones (Table 2) were reduced in dichloromethane, and the molar ratio of ketone to **1** was 1/1. Under the standard conditions for reduction of aldehydes (25 °C, 1 equiv acetic acid), the reaction of 1-(4-bromophenyl)ethanone (**19**) was very slow. Heating of **19** (1 equiv), **1** (1 equiv) and acetic acid (1 equiv) at 40 °C for 24 h gave a moderate yield (68%) of secondary alcohol **26**, but unreacted ketone remained. This problem was solved by increasing the amount of acetic acid to 5 equiv. Now ketone **19** was consumed in 24 h and alcohol **26** was isolated in 93% yield (Table 2, entry 1). The excess of acetic acid was removed prior to chromatography during evaporation, so the simple isolation procedure was not changed. Under these conditions, ketones **20–25** were reduced to the corresponding secondary alcohols **27–33** in good yields (Table 2). Reductions of 4-*tert*-butylcyclohexanone (**21**) and 4-phenyl-2-butanone (**22**) needed only 1 equiv of acetic acid (as with aldehydes), while the other substrates were reduced with 5 equiv.

**Table 2:** Reductions of ketones by diMe-Imd-BH_3_ (**1**) and acetic acid.^a^

entry	ketone	alcohol	yield

1	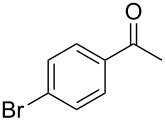 **19**	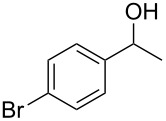 **26**	93%
2	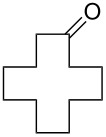 **20**	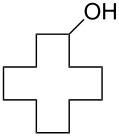 **27**	88%
3	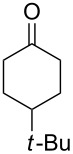 **21**	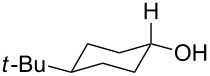 **28** 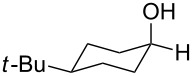 **29**	65%^b^ 24%^b^
4	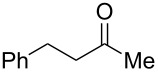 **22**	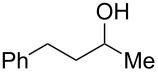 **30**	88%^b^
5	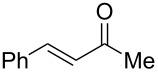 **23**	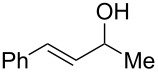 + **30** **31**	79%^c^ (90:10)
6	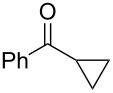 **24**	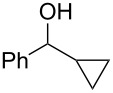 **32**	83%^d^
7	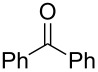 **25**	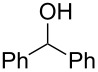 **33**	97%^e^

^a^Conditions: ketone (0.5 mmol), **1** (0.5 mmol), and AcOH (2.5 mmol) in CH_2_Cl_2_ (2 mL) at 40 °C for 24 h; ^b^only 1 equiv of acetic acid was used; ^c^minor product **30** could not be separated; ^d^reaction time was 48 h; ^e^reaction time was 72 h.

Reduction of 4-*tert*-butylcyclohexanone (**21**) gave the *cis*-alcohol **28** in 65% yield and the *trans*-alcohol in 24% yield (89% combined yield). The major product results from axial attack and is seen with borohydride reagents [26–27] as well as with the combination of **1** and silica gel. Reaction of (*E*)-4-phenylbut-3-en-2-one (**23**) gave the 1,2-reduction product **31** along with a small amount of doubly reduced, saturated alcohol **30** (90:10). The combined yield of these inseparable products was 79%. Reductions of hindered ketones **24** and **25** were slower even with 5 equiv of acetic acid, but gave the corresponding alcohols **32** and **33** in 83% and 97% after 48–72 h. For comparison, the yield of the reaction of **24** using silica gel was 73% after 96 h [20]. Overall, the reductions of aldehydes and ketones with the combination of **1** and acetic acid gave comparable yields to the silica procedure.

**Scheme 3:**
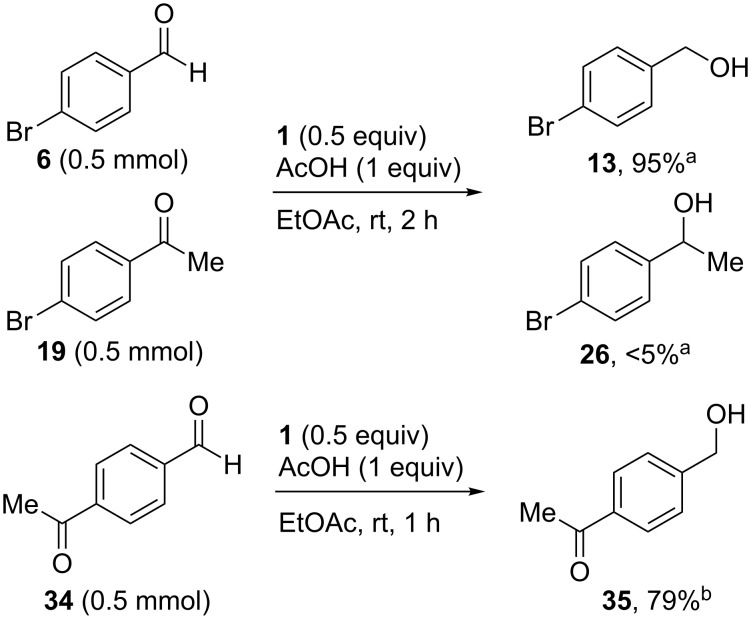
Chemoselective reductions of aldehydes with **1** and acetic acid. ^a^Determined by 1H NMR spectroscopic analysis of the crude product; ^b^isolated yield after flash chromatography.

Chemoselective reductions were also readily achieved under the present reaction conditions (Scheme 3). Treatment of a mixture of aldehyde **6** (0.5 mmol) and ketone **19** (0.5 mmol) with **1** (0.25 mmol) and acetic acid (1 equiv) in EtOAc exclusively provided the primary alcohol **13** in 95% yield**,** while the ketone **19** was intact (<5% conversion to **26**). The reduction of 4-acetylbenzaldehyde (**34**) was also chemoselective and afforded only 4-acetylbenzyl alcohol (**35**) in 79% yield.

## Conclusion

In summary, we have shown that acetic acid is a good activator for the reductions of aldehydes and ketones by stable NHC-borane **1**. DiMe-Imd-BH_3_ (**1**), and by implication related carbene-boranes, are convenient reagents for small-scale reductions, because their ease of handling allows simple reaction and separation procedures.

The results of control experiments suggest that the acetic acid activates the carbonyl component, not the NHC-borane. Thus, the acid does not consume a hydride equivalent of NHC-BH_3_, and a stoichiometric amount of H_2_ gas (a safety hazard on a large scale) is not produced.

Further research and process-development work are needed before one can conclude that diMe-Imd-BH_3_ and related carbene boranes are generally attractive reagents on larger scales [5,21]. However, based on the established physical and chemical properties of **1** and its relatives and on the convenience of the small-scale reduction procedures with both silica gel and acetic acid, NHC-boranes deserve serious consideration as candidates for large-scale reductions.

## Experimental

**Procedure for aldehyde reduction** (examples in Scheme 2, Scheme 3, and Table 1): diMe-Imd-BH_3_ (**1**, 27.5 mg, 0.25 mmol) and acetic acid (30.0 mg, 0.50 mmol) were added to a solution of 4-bromobenzaldehyde (**6**, 92.5 mg, 0.50 mmol) in EtOAc (2 mL). After 24 h at room temperature, the solvent was removed under reduced pressure, and the residue was purified by silica-gel flash chromatography (hexane/EtOAc, 1:1) to give 4-bromophenylmethanol (**13**, 74.0 mg, 79%) as a white solid.

**^11^B NMR experiments in** Figure 1**, entries 4–6:** The above procedure was followed on the same scale but with varying amounts of **1**. After the solvent was removed, the residue was taken up in C_6_D_6_ prior to recording of the ^11^B NMR spectrum.

**Procedure for ketone reduction** (examples in Table 2): DiMe-Imd-BH_3_ (**1**, 55.0 mg, 0.50 mmol) and acetic acid (150 mg, 2.5 mmol) were added to a solution of 1-(4-bromophenyl)ethanone (**19**, 99.6 mg, 0.50 mmol) in CH_2_Cl_2_ (2 mL). After stirring for 24 h at 40 °C, the mixture was cooled, and the solvent was removed under reduced pressure. The residue was purified by silica-gel flash chromatography (hexane/EtOAc, 2:1) to give 1-(4-bromophenyl)ethanol (**26**, 94.2 mg, 93%) as a colorless oil.

## Supporting Information

File 1 contains copies of the ^1^H NMR spectra of all the products from Figure 1, Scheme 2 and Scheme 3, and Table 1 and Table 2.
NMR spectra of all products.

## References

[1] Burkhardt E R, Matos K (2006). Chem Rev.

[2] Lane C F (2005). Aldrichimica Acta.

[3] Seyden-Penne J (1991). Reductions by the Aluminohydrides and Borohydrides in Organic Synthesis.

[4] Chen G, Kaufmann D E, Matteson D S (2004). Boron Compounds. Organometallics.

[5] Atkins W J, Burkhardt E R, Matos K (2006). Org Process Res Dev.

[6] Curran D P, Solovyev A, Makhlouf Brahmi M, Fensterbank L, Malacria M, Lacôte E (2011). Angew Chem, Int Ed.

[7] Wang Y, Robinson G H (2011). Inorg Chem.

[8] Solovyev A, Chu Q, Geib S J, Fensterbank L, Malacria M, Lacôte E, Curran D P (2010). J Am Chem Soc.

[9] Carboni B, Monnier L (1999). Tetrahedron.

[10] Carboni B, Carreaux F, Kaufmann D E, Matteson D S (2004). Boron Compounds. Organometallics.

[11] Staubitz A, Robertson A P M, Sloan M E, Manners I (2010). Chem Rev.

[12] Lindsay D M, McArthur D (2010). Chem Commun.

[13] Chu Q, Makhlouf Brahmi M, Solovyev A, Ueng S-H, Curran D P, Malacria M, Fensterbank L, Lacôte E (2009). Chem–Eur J.

[14] Horn M, Mayr H, Lacôte E, Merling E, Deaner J, Wells S, McFadden T, Curran D P (2012). Org Lett.

[15] Dorothea R, Herbert M (2009). Angew Chem, Int Ed.

[16] Mayr H, Ofial A R (2008). J Phys Org Chem.

[17] Mayr H, Bug T, Gotta M F, Hering N, Irrgang B, Janker B, Kempf B, Loos R, Ofial A R, Remennikov G (2001). J Am Chem Soc.

[18] Funke M A, Mayr H (1997). Chem–Eur J.

[19] Mayr H, Patz M (1994). Angew Chem, Int Ed Engl.

[20] Taniguchi T, Curran D P (2012). Org Lett.

[21] Abdel-Magid A F, Carson K G, Harris B D, Maryanoff C A, Shah R D (1996). J Org Chem.

[22] Gribble G W, Ferguson D C (1975). J Chem Soc, Chem Commun.

[R23] 23These NMR experiments were conducted in C_6_D_6_ to avoid water and for comparison with known resonances.

[24] Solovyev A, Geib S J, Lacôte E, Curran D P (2012). Organometallics.

[R25] 25A precipitate formed in some reactions. This is presumably an imidazolium salt derived from the NHC that is released on formation of B(OR)_3_, but it could also contain insoluble boron species.

[26] Lansbury P T, MacLeay R E (1963). J Org Chem.

[27] Wigfield D C (1979). Tetrahedron.

