# Supplementary material for: Reductions of aldehydes and ketones with a readily available N-heterocyclic carbene borane and acetic acid
Source: Beilstein J Org Chem. 2013 Apr 8;9:675–80. doi: 10.3762/bjoc.9.76 (PMC3629027; doi:10.3762/bjoc.9.76)
Supplement: File 1 — contains copies of the 1H NMR spectra of all the products from Figure 1, Scheme 2 and Scheme 3, and Table 1 and Table 2. NMR spectra of all products. [file Beilstein_J_Org_Chem-09-675-s001.pdf]

Supporting Information

for

Reductions of aldehydes and ketones with a readily available N-heterocyclic carbene borane and acetic acid

Vladimir Lamm, Xiangcheng Pan, Tsuyoshi Taniguchi*[§] and Dennis P. Curran*

Address: Department of Chemistry, University of Pittsburgh, Pittsburgh, PA 15260 USA

Email: Tsuyoshi Taniguchi* - tsuyoshi@p.kanazawa-u.ac.jp; Dennis P. Curran* - curran@pitt.edu

* Corresponding author

[§] Current address: School of Pharmaceutical Sciences, Institute of Medical, Pharmaceutical and Health Sciences, Kanazawa University, Kakuma-machi, Kanazawa 920-1192, Japan

NMR spectra of all products

Table of contents

Copies of ^{11}B NMR spectra of boron products from Figure 1 .... S2

Copies of ^{1}H NMR spectra of isolated reduction products S7

The experimental procedures for aldehyde and ketone reductions are described at the end of the paper. All of the resulting alcohols are known compounds. The ^{1}H NMR spectra of the isolated alcohols (after automated flash chromatography) are included to show the quality of the products obtained from the reductions in Table 1, Table 2, and Scheme 3.

XP-98-43,1 d, 11B, C6D6, 500, 2/7/2012

Figure 1, entry 2

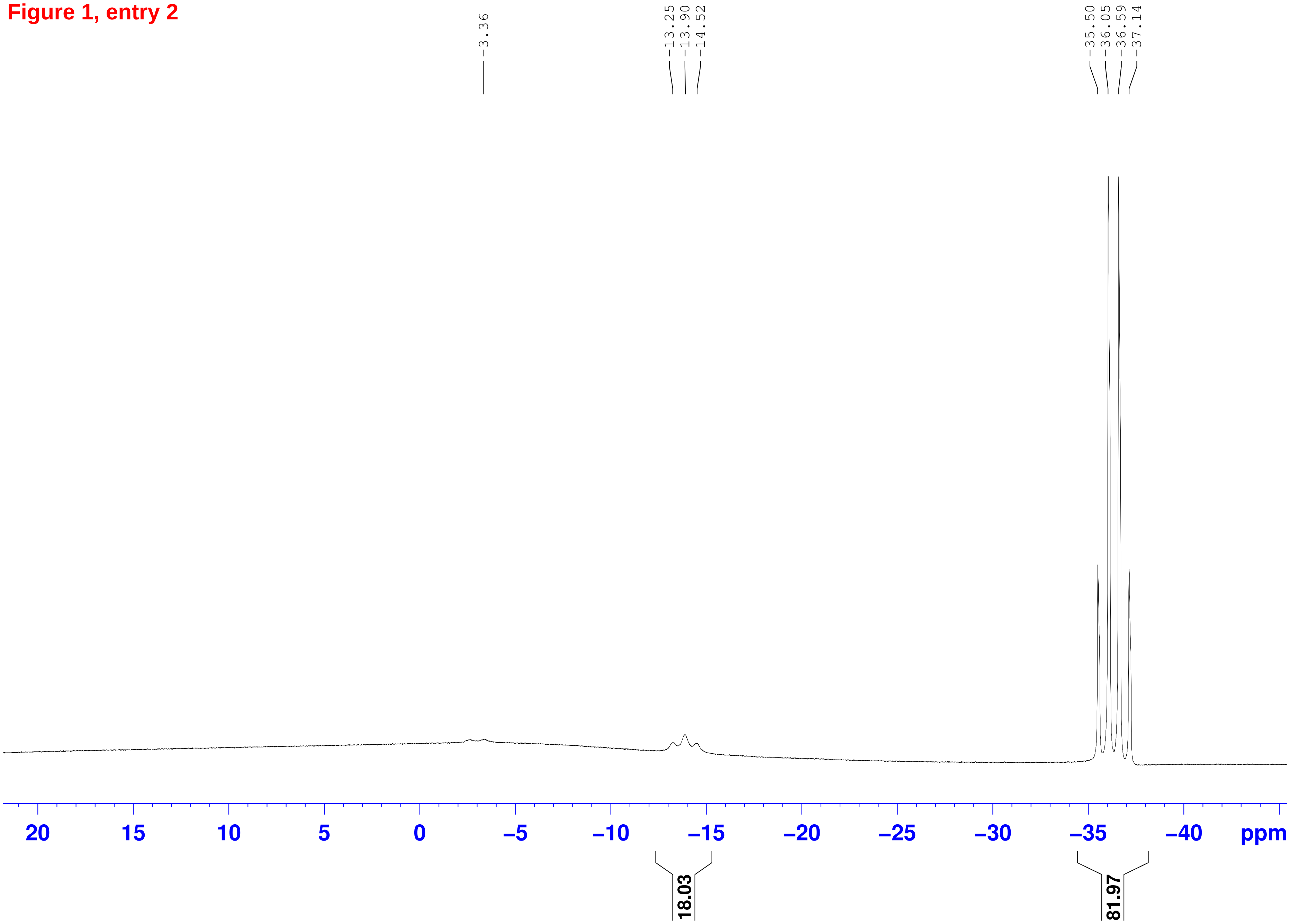

XP-98-43,3 d, 11B, C6D6, 500, 2/10/2012

Figure 1, entry 3

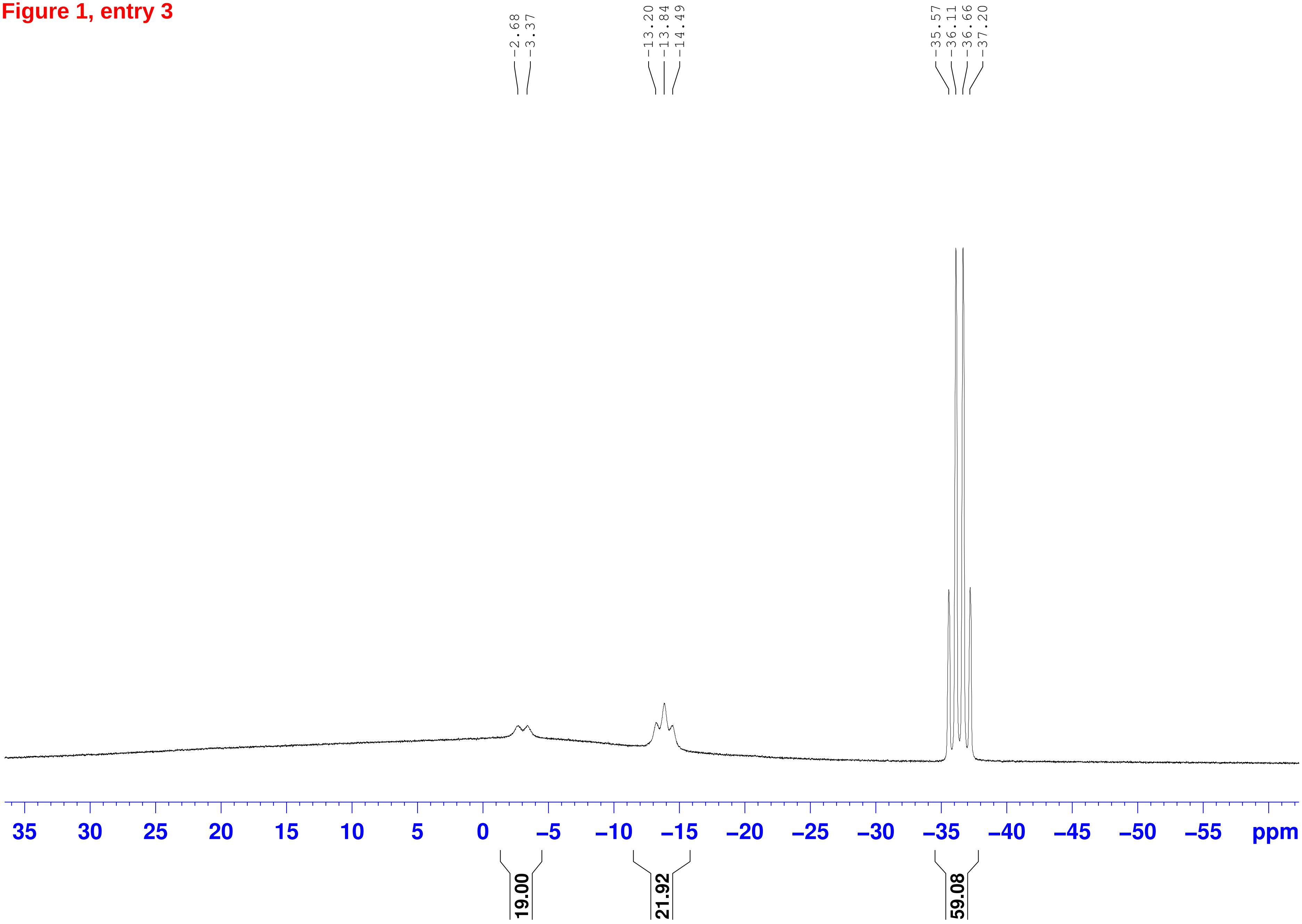

XP-118-81, C6D6, 400A, 1/21/2013

Figure 1, entry 4

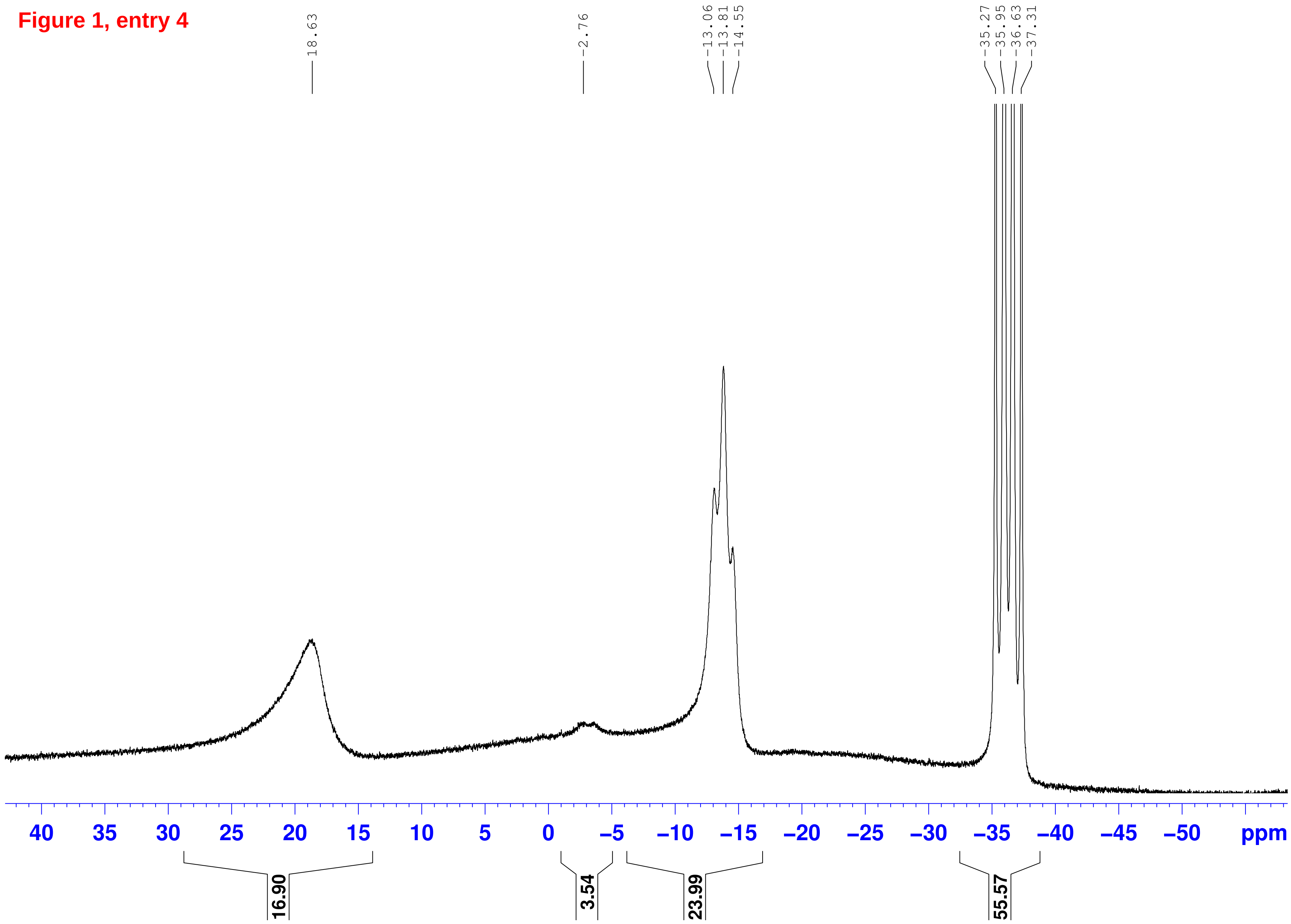

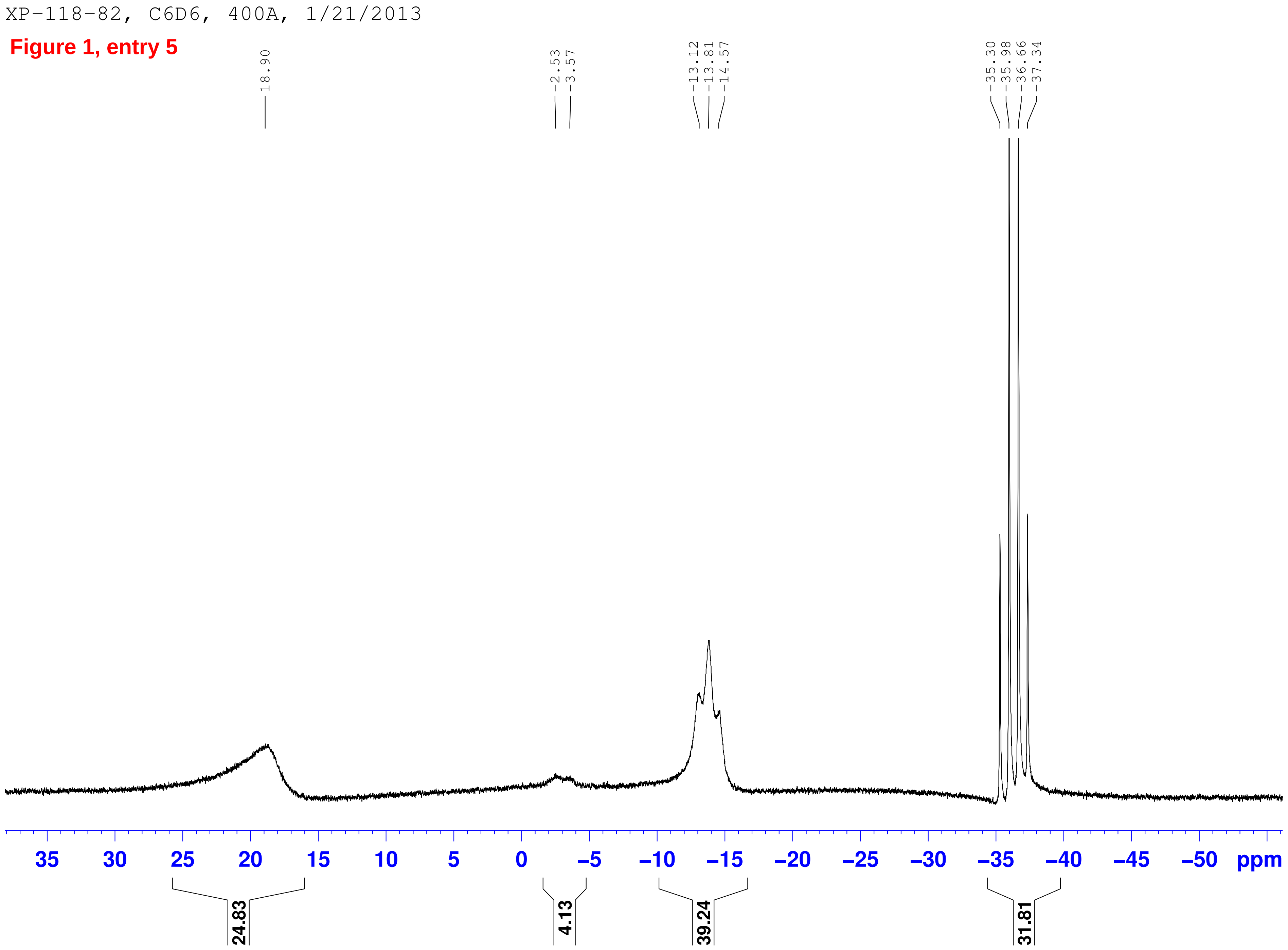
XP-118-82, C6D6, 400A, 1/21/2013
Figure 1, entry 5
18.90
-2.53
-3.57
-13.12
-13.81
-14.57
-35.30
-35.98
-36.66
-37.34
35 30 25 20 15 10 5 0 −5 −10 −15 −20 −25 −30 −35 −40 −45 −50 ppm
24.83
4.13
39.24
31.81

XP-118-83, 11B, C6D6, 400A, 1/21/2013

Figure 1, entry 6

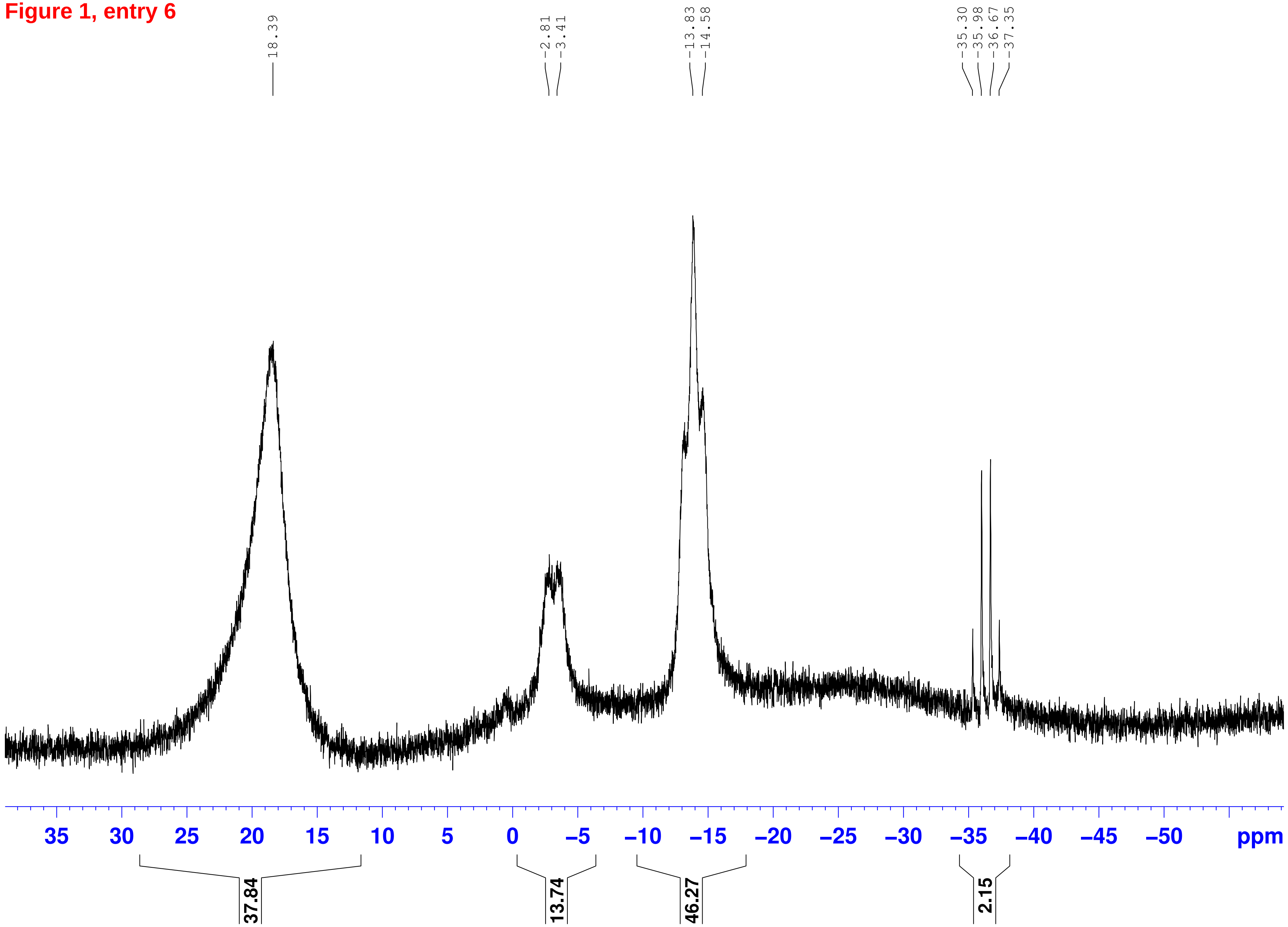

VL-39-70

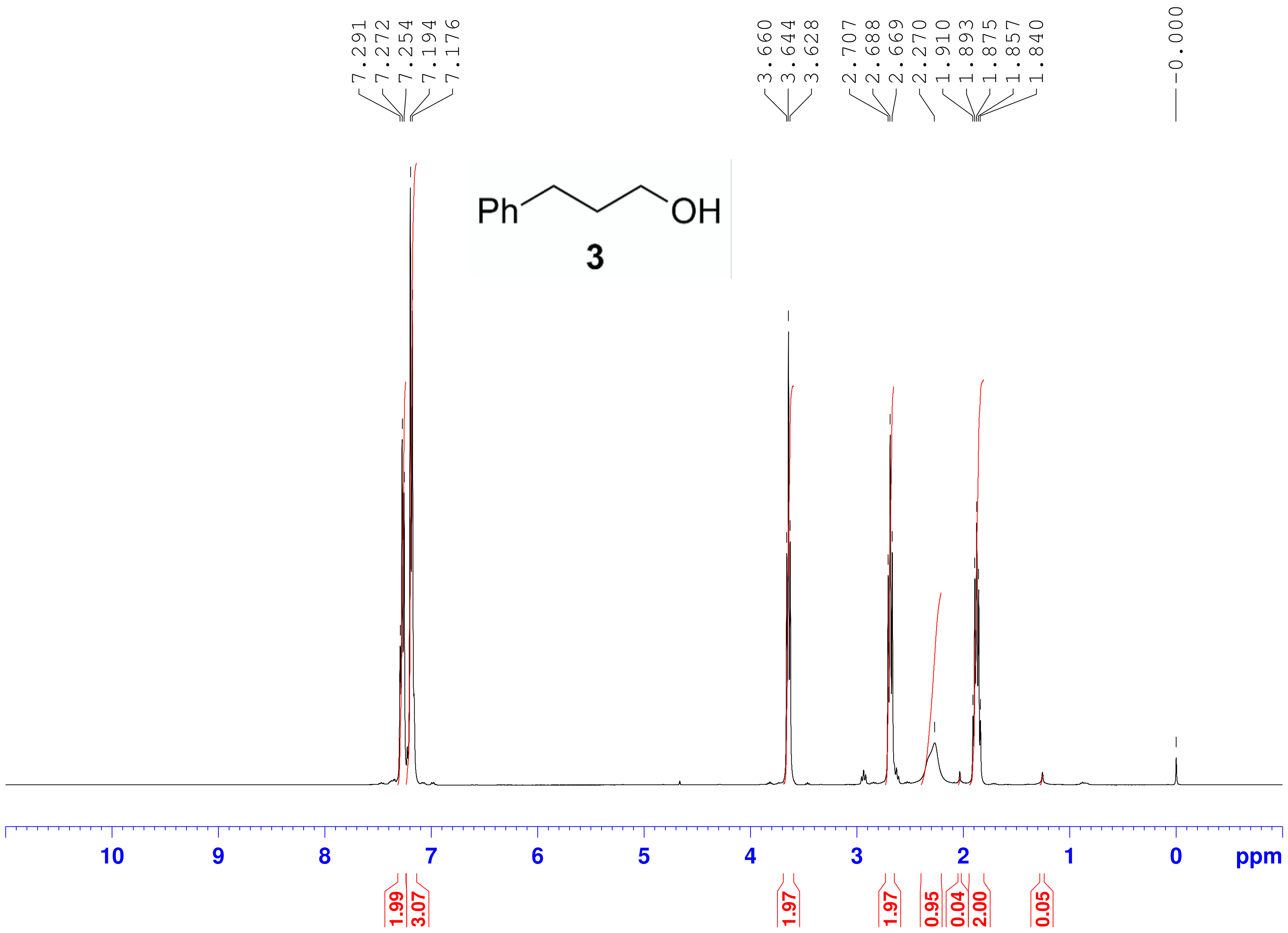
7.291
7.272
7.254
7.194
7.176
3.660
3.644
3.628
2.707
2.688
2.669
2.270
1.910
1.893
1.875
1.857
1.840
-0.000
Ph
OH
3
10
9
8
7
6
5
4
3
2
1
0
ppm
1.99
3.07
1.97
1.97
0.95
0.04
2.00
0.05

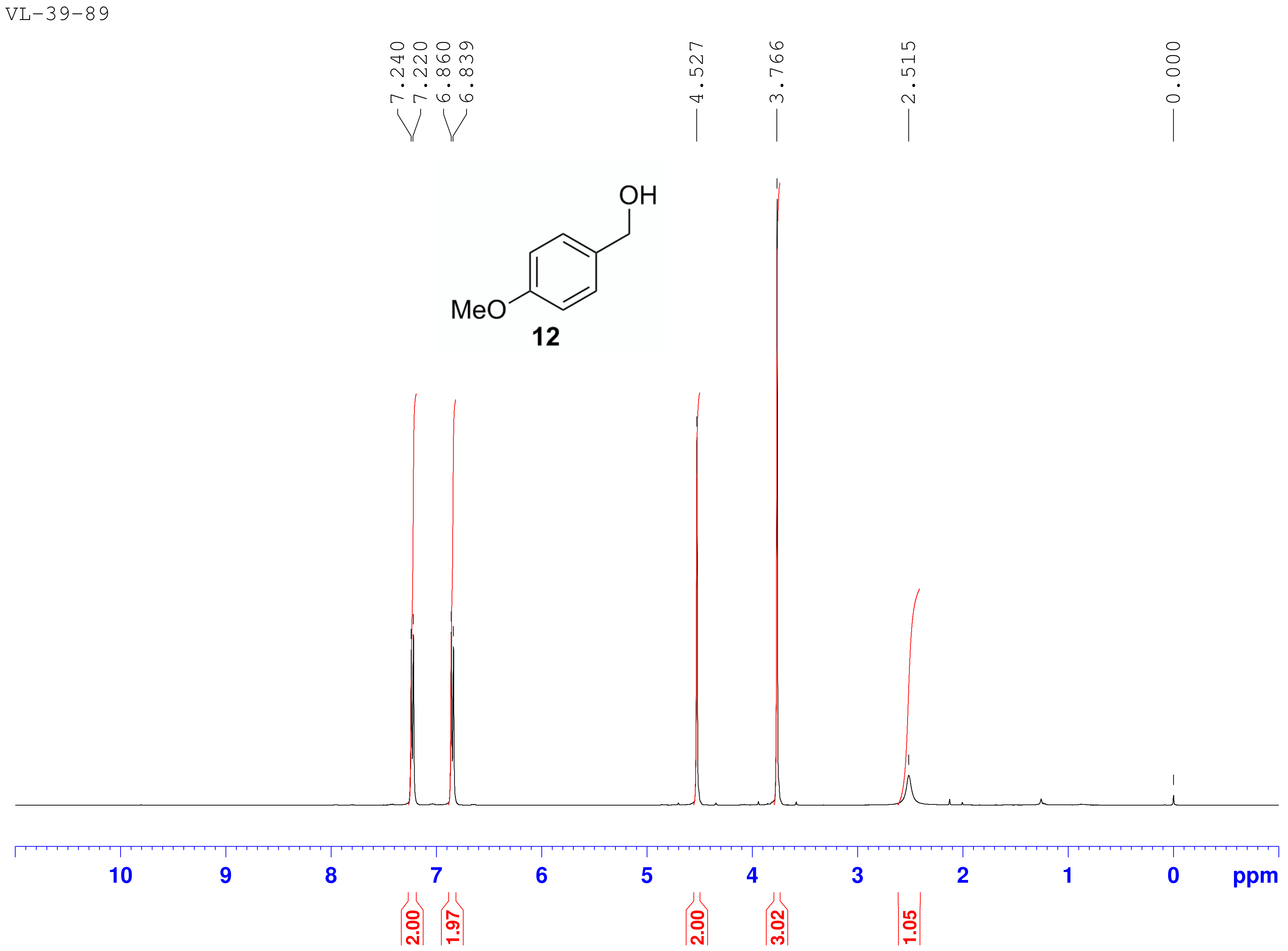
VL-39-89
7.240
7.220
6.860
6.839
4.527
3.766
2.515
0.000
OH
MeO
12
10
9
8
7
6
5
4
3
2
1
0
ppm
2.00
1.97
2.00
3.02
1.05

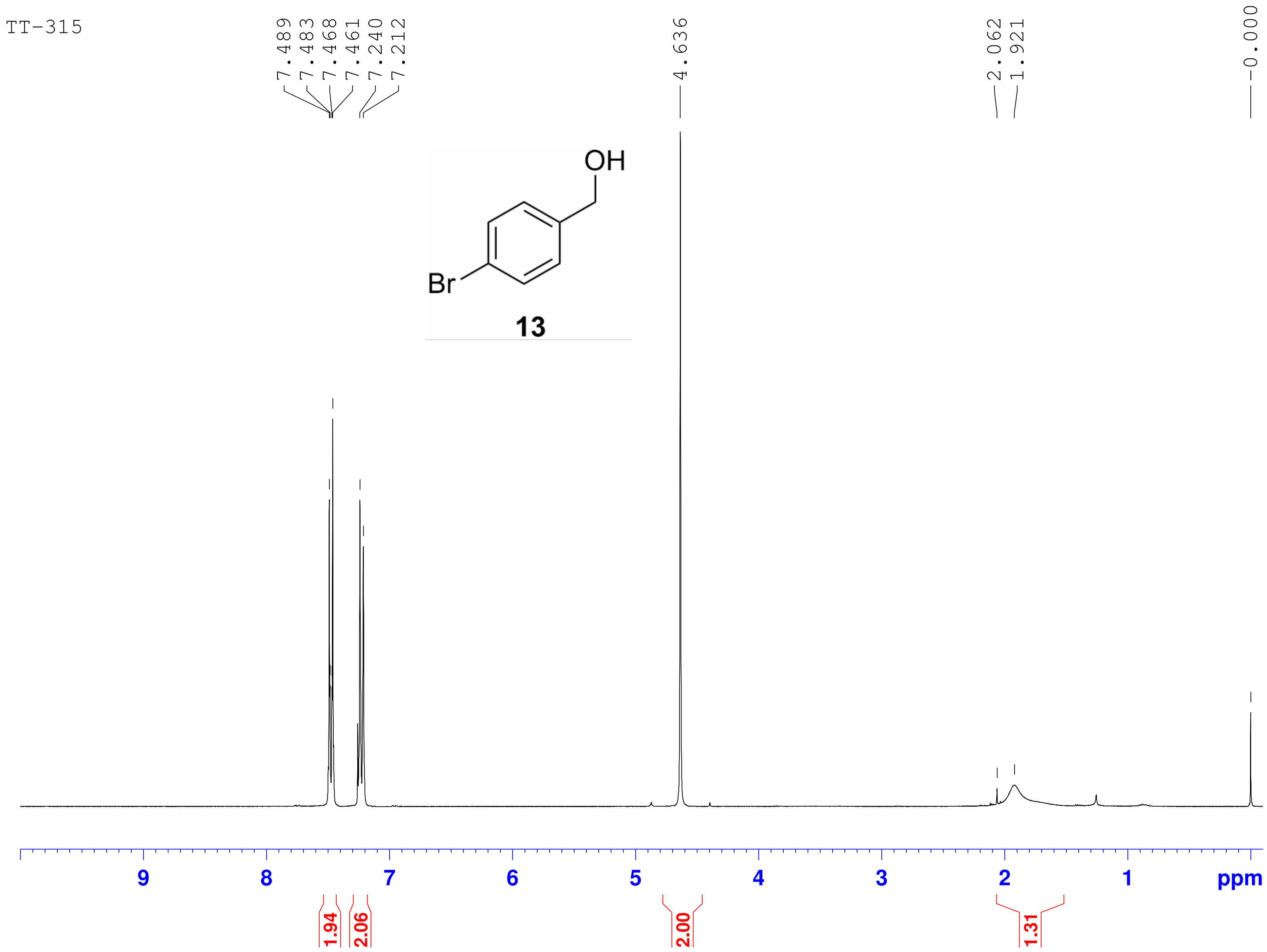
TT-315
7.489
7.483
7.468
7.461
7.240
7.212
4.636
2.062
1.921
0.000
OH
Br
13
9
8
7
6
5
4
3
2
1
ppm
1.94
2.06
2.00
1.31

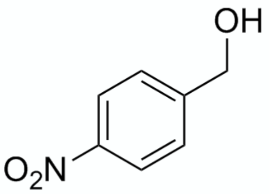
OH
O2N

14

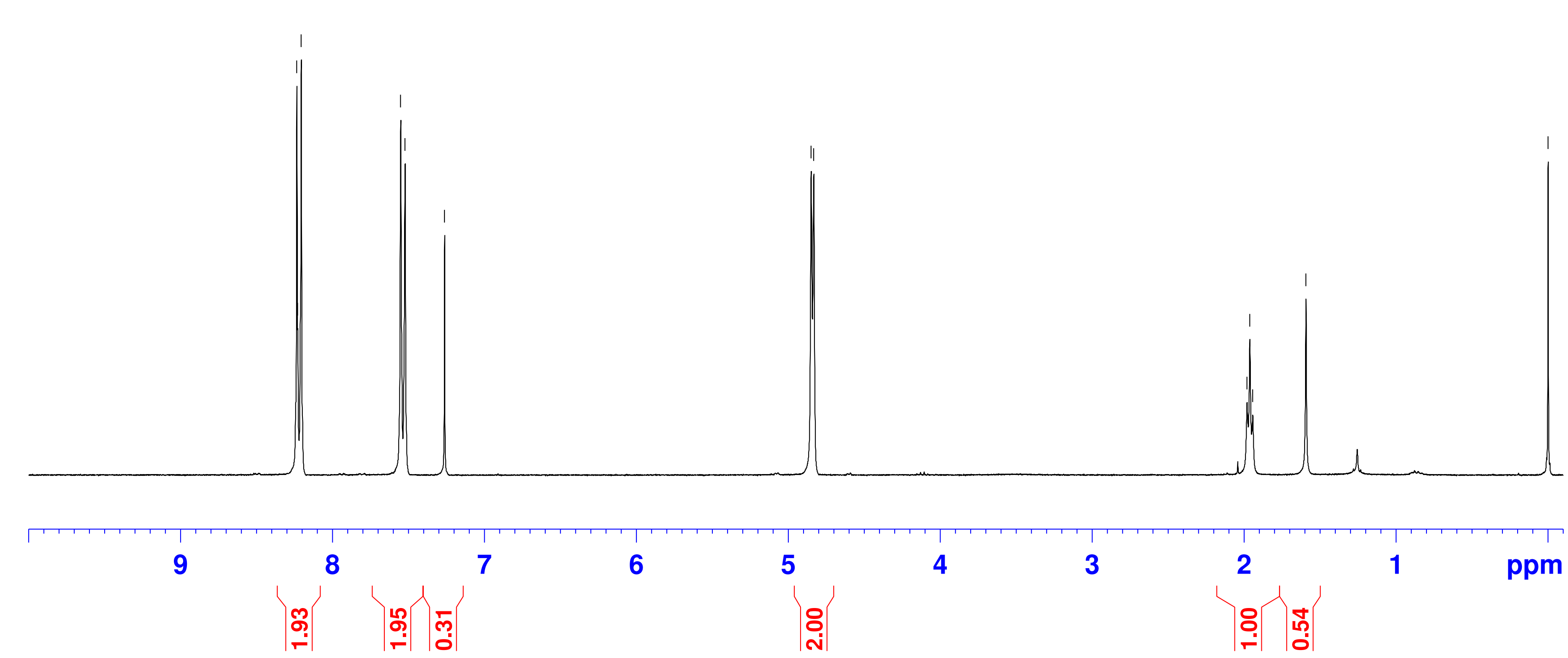
TT-313
8.236
8.230
8.207
7.553
7.523
7.263
4.851
4.833
1.982
1.963
1.944
1.594
-0.000
9
8
7
6
5
4
3
2
1
ppm
1.93
1.95
0.31
2.00
1.00
0.54

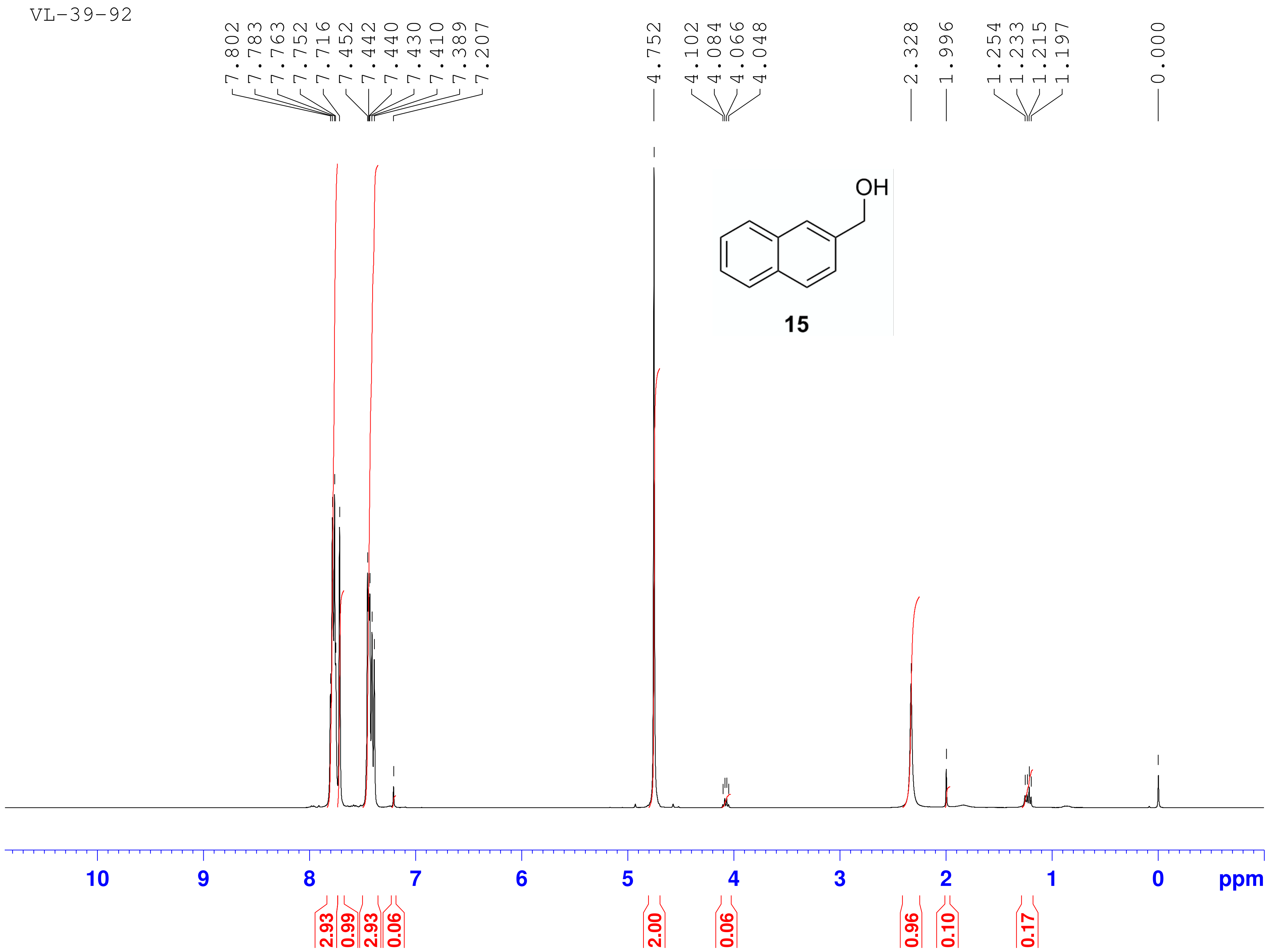
VL-39-92
7.802
7.783
7.763
7.752
7.716
7.452
7.442
7.440
7.430
7.410
7.389
7.207
4.752
4.102
4.084
4.066
4.048
2.328
1.996
1.254
1.233
1.215
1.197
0.000
OH
15
10
9
8
7
6
5
4
3
2
1
0
ppm
2.93
0.99
2.93
0.06
2.00
0.06
0.96
0.10
0.17

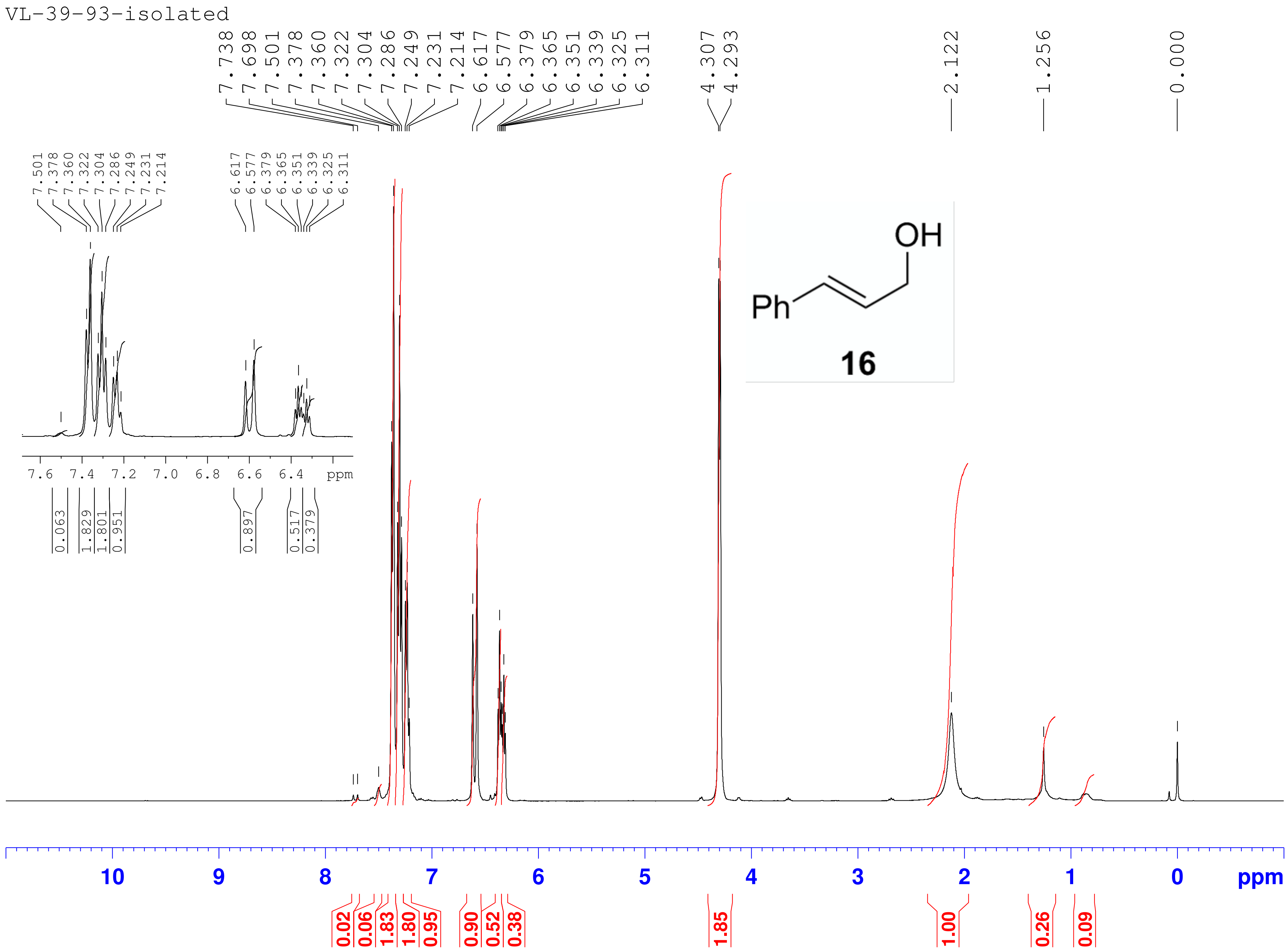
VL-39-93-isolated
7.738
7.698
7.501
7.378
7.360
7.322
7.304
7.286
7.249
7.231
7.214
6.617
6.577
6.379
6.365
6.351
6.339
6.325
6.311
4.307
4.293
2.122
1.256
0.000
OH
Ph
16
7.6 7.4 7.2 7.0 6.8 6.6 6.4 ppm
0.063
1.829
1.801
0.951
0.897
0.517
0.379
10 9 8 7 6 5 4 3 2 1 0 ppm
0.02
0.06
1.83
1.80
0.95
0.90
0.52
0.38
1.85
1.00
0.26
0.09

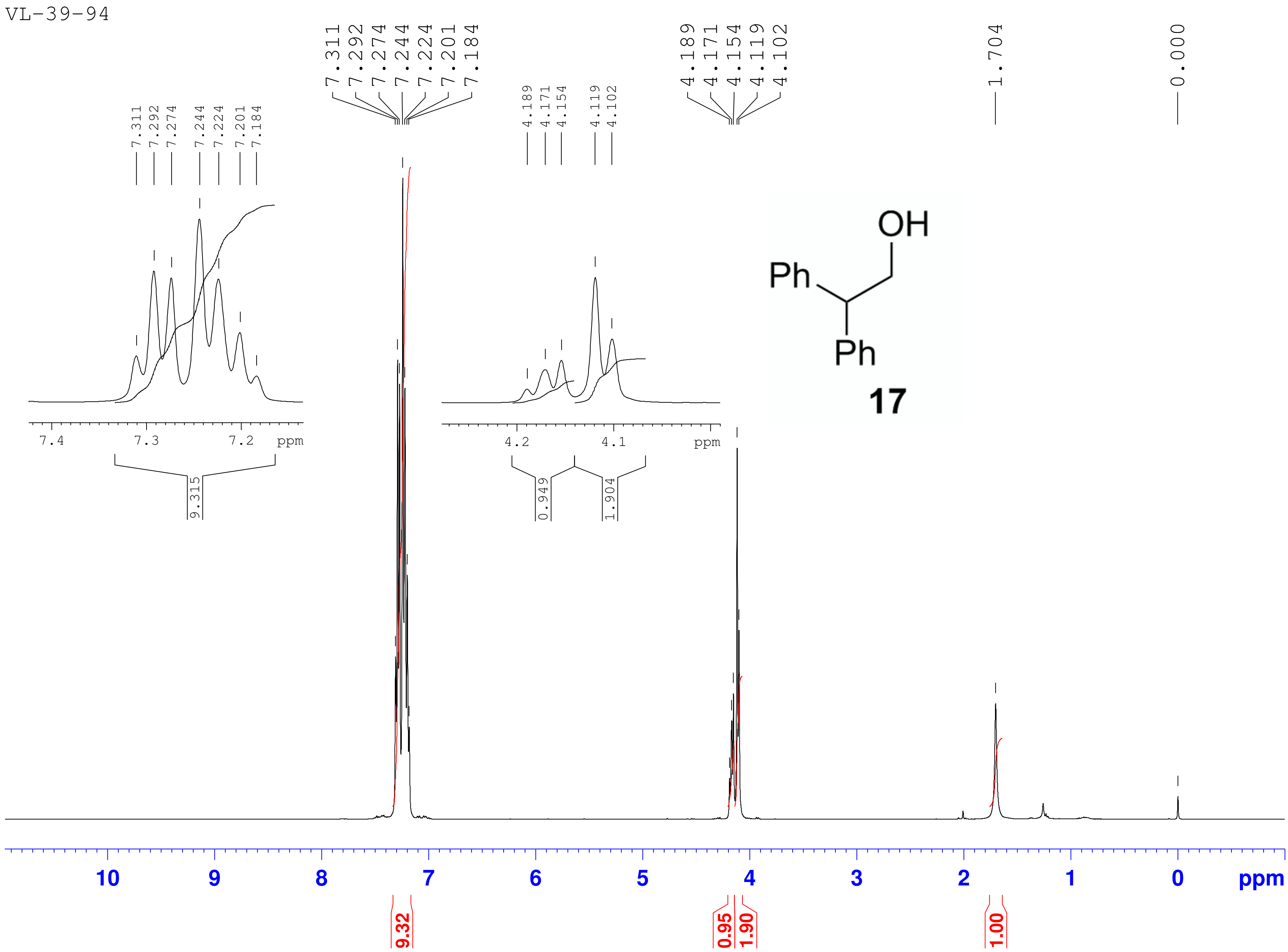
VL-39-94
7.311
7.292
7.274
7.244
7.224
7.201
7.184
4.189
4.171
4.154
4.119
4.102
1.704
0.000
7.4
7.3
7.2
ppm
9.315
4.2
4.1
ppm
0.949
1.904
OH
Ph
Ph
17
10
9
8
7
6
5
4
3
2
1
0
ppm
9.32
0.95
1.90
1.00

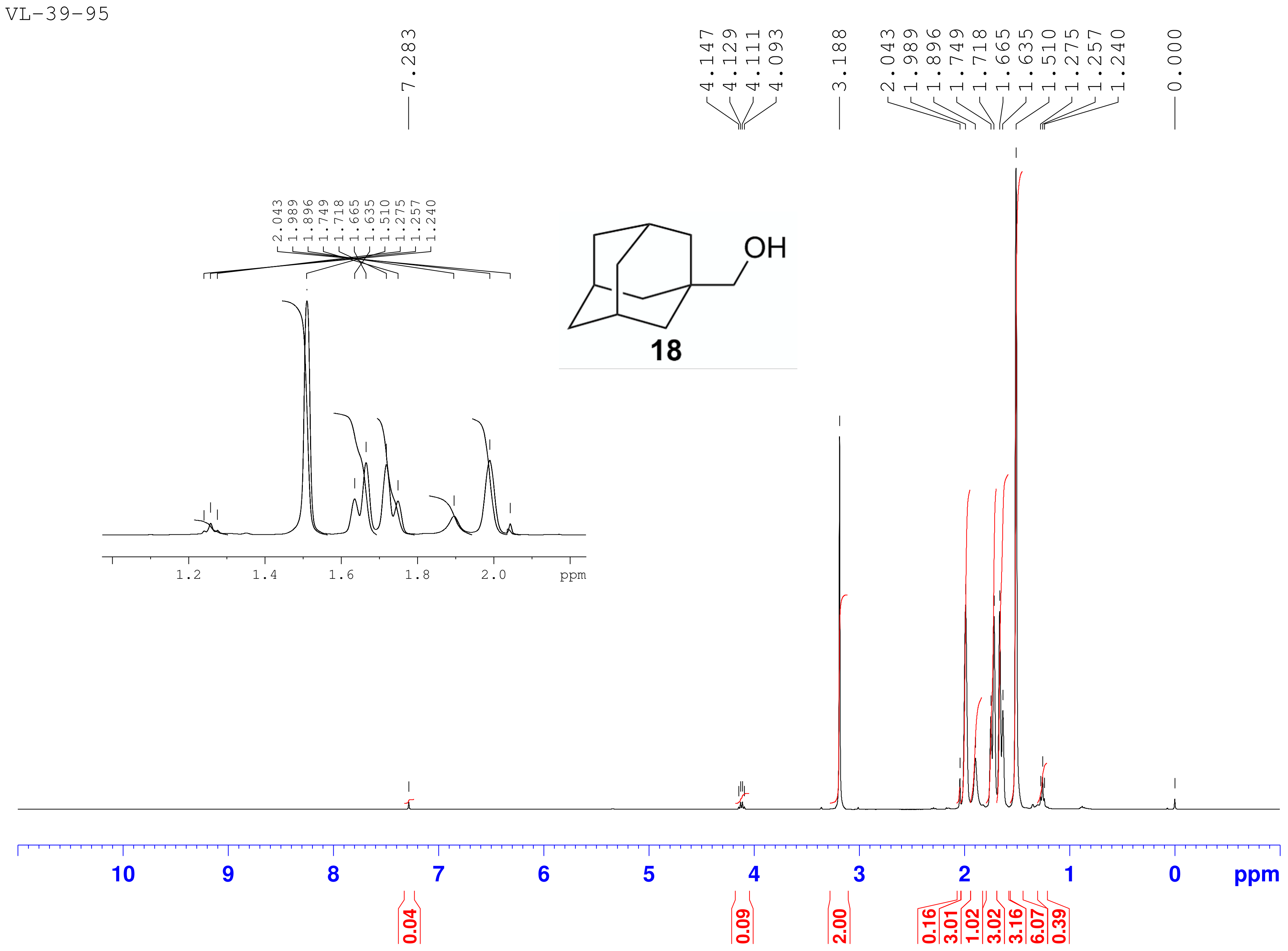
VL-39-95
7.283
4.147
4.129
4.111
4.093
3.188
2.043
1.989
1.896
1.749
1.718
1.665
1.635
1.510
1.275
1.257
1.240
0.000
OH
18
1.2
1.4
1.6
1.8
2.0
ppm
10
9
8
7
6
5
4
3
2
1
0
ppm
0.04
0.09
2.00
0.16
3.01
1.02
3.02
3.16
6.07
0.39

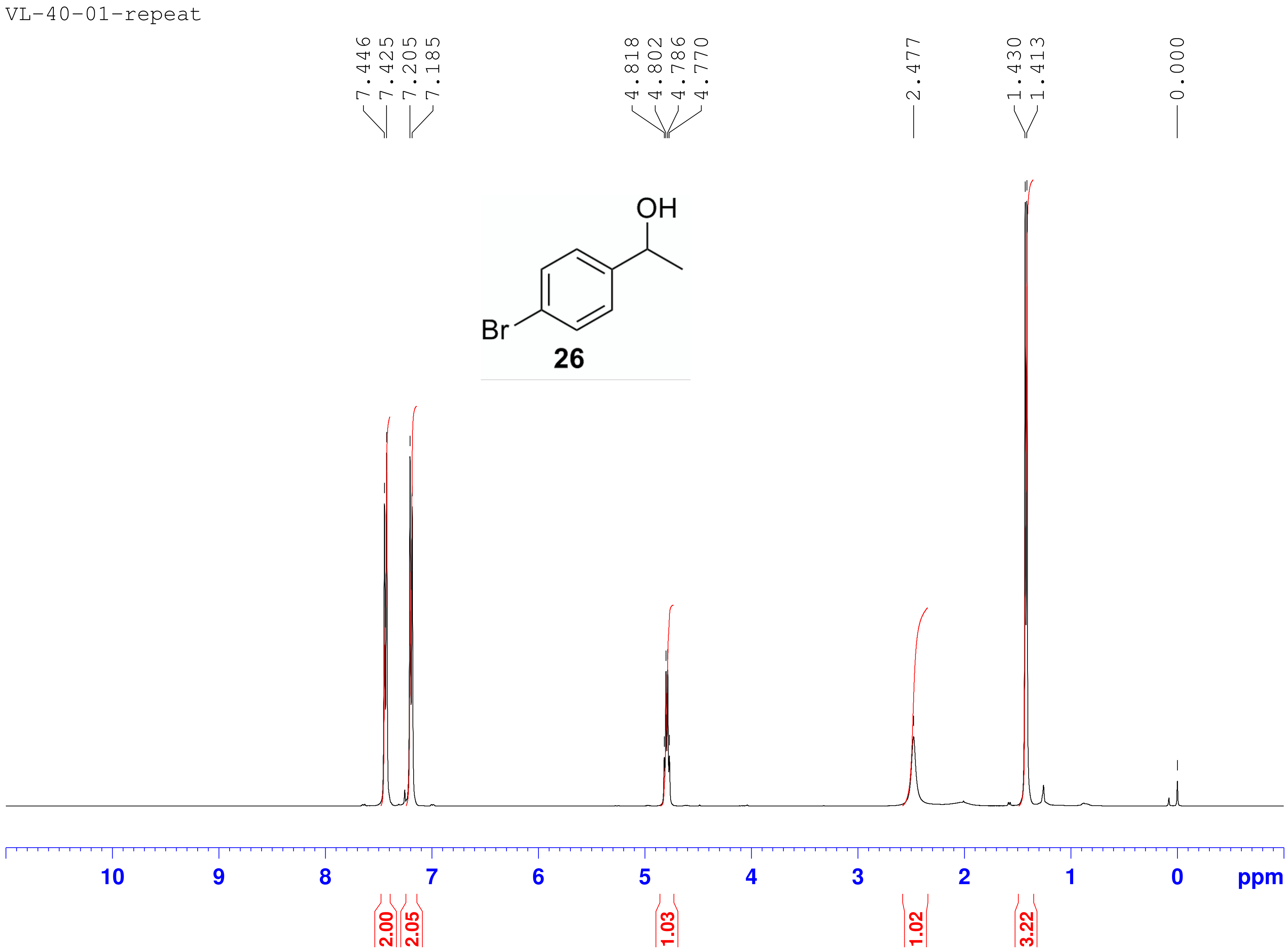
VL-40-01-repeat
7.446
7.425
7.205
7.185
4.818
4.802
4.786
4.770
2.477
1.430
1.413
0.000
OH
Br
26
10
9
8
7
6
5
4
3
2
1
0
ppm
2.00
2.05
1.03
1.02
3.22

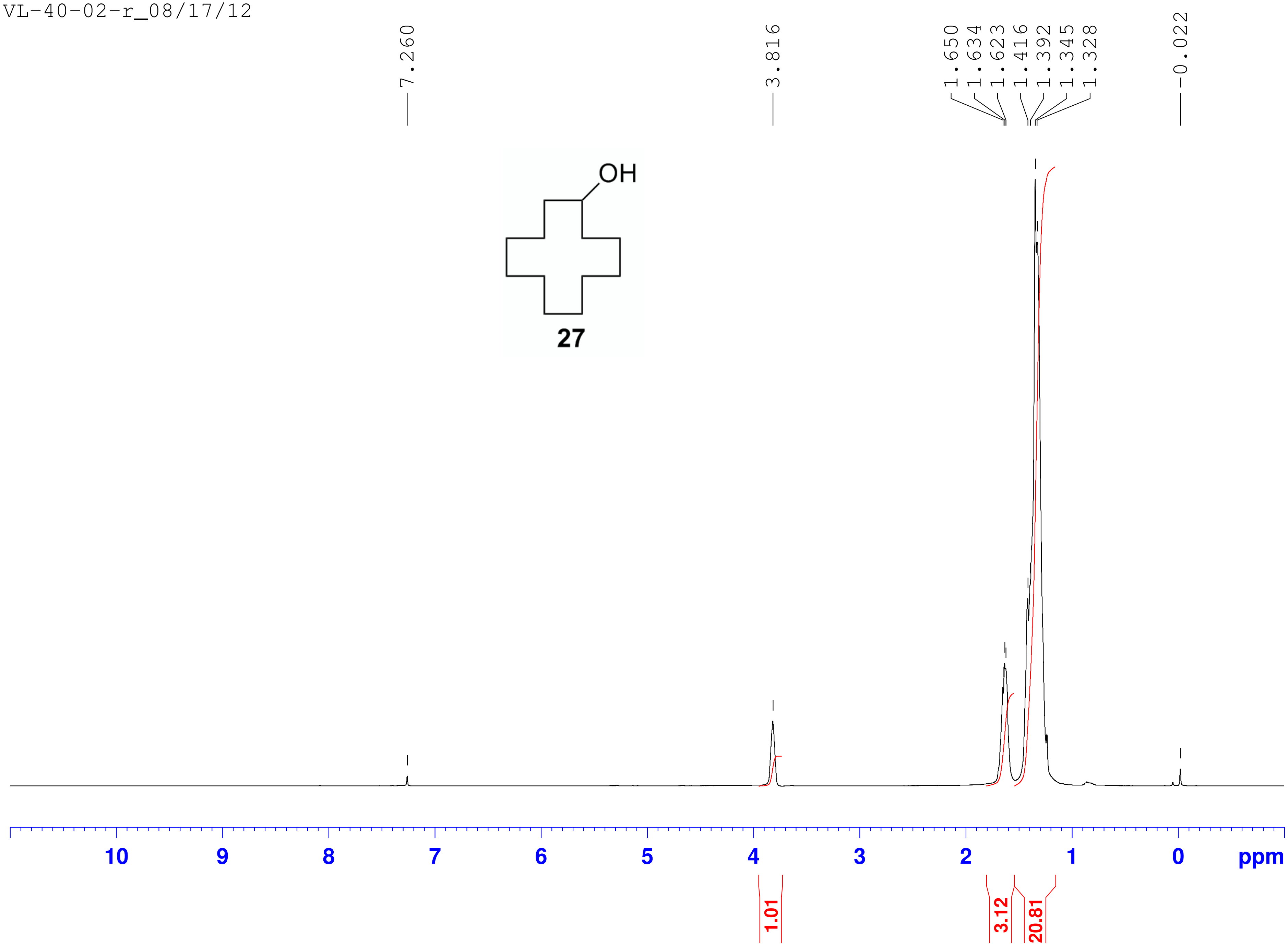
VL-40-02-r_08/17/12
7.260
3.816
1.650
1.634
1.623
1.416
1.392
1.345
1.328
-0.022
OH
27
10
9
8
7
6
5
4
3
2
1
0
ppm
1.01
3.12
20.81

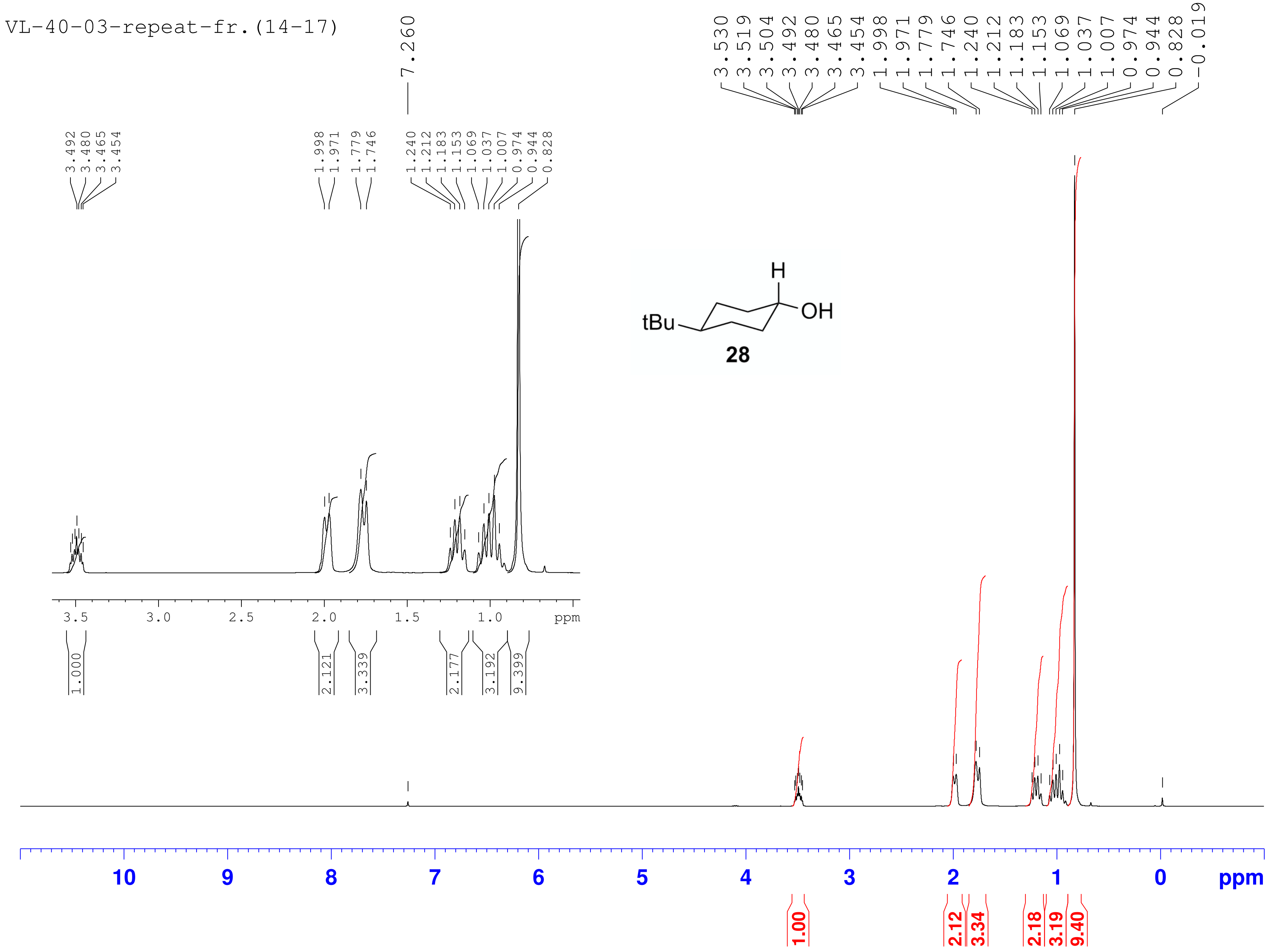
VL-40-03-repeat-fr.(14-17)
7.260
3.530 3.519 3.504 3.492 3.480 3.465 3.454 1.998 1.971 1.779 1.746 1.240 1.212 1.183 1.153 1.069 1.037 1.007 0.974 0.944 0.828 -0.019
3.492 3.480 3.465 3.454
1.998 1.971 1.779 1.746
1.240 1.212 1.183 1.153 1.069 1.037 1.007 0.974 0.944 0.828
3.5 3.0 2.5 2.0 1.5 1.0 ppm
1.000 2.121 3.339 2.177 3.192 9.399
H
OH
tBu
28
10 9 8 7 6 5 4 3 2 1 0 ppm
1.00 2.12 3.34 2.18 3.19 9.40

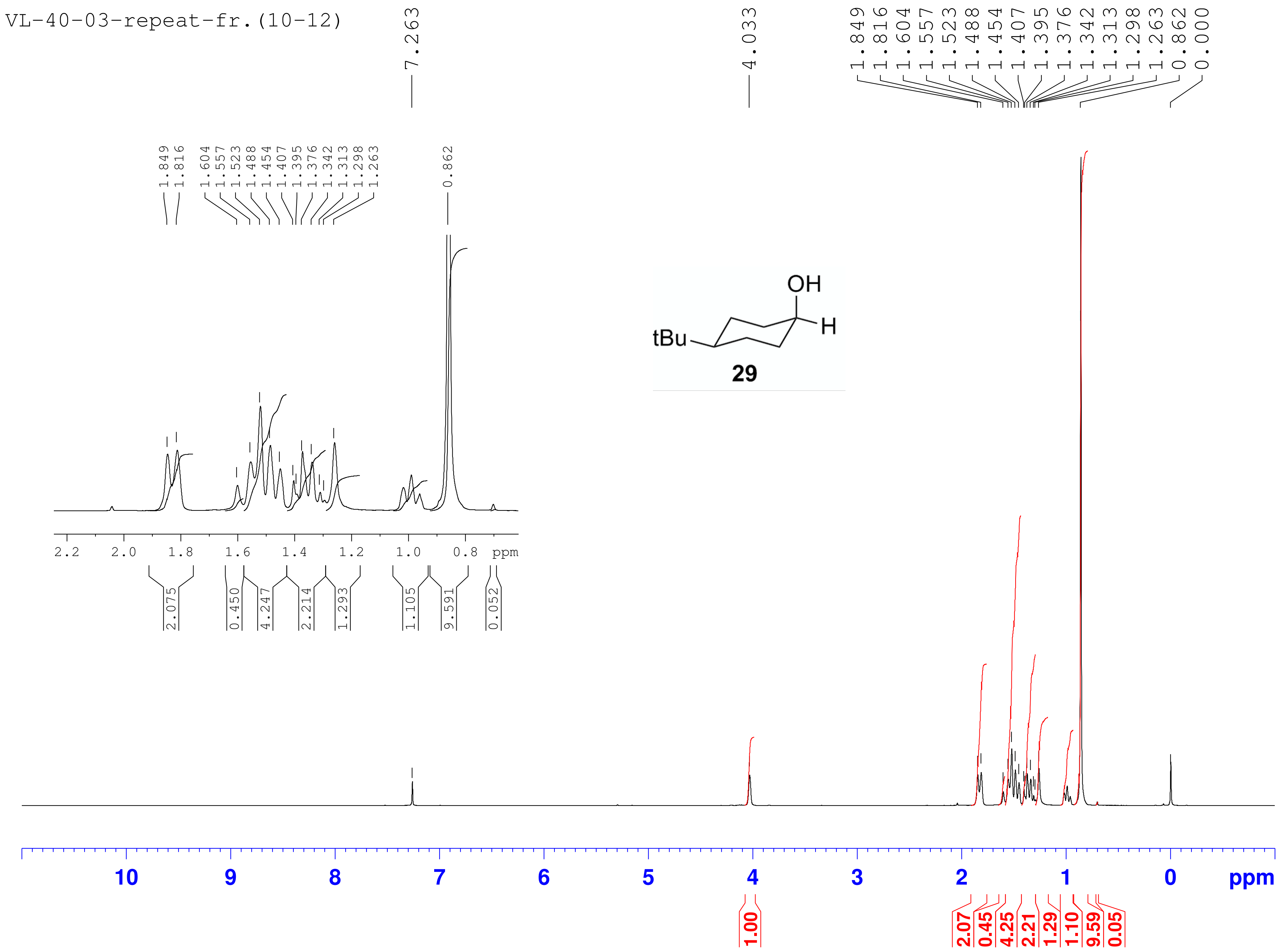
VL-40-03-repeat-fr.(10-12)
7.263
4.033
1.849
1.816
1.604
1.557
1.523
1.488
1.454
1.407
1.395
1.376
1.342
1.313
1.298
1.263
0.862
0.000
OH
H
tBu
ppm
1.00
2.07
0.45
4.25
2.21
1.29
1.10
9.59
0.05
2.075
0.450
4.247
2.214
1.293
1.105
9.591
0.052

29

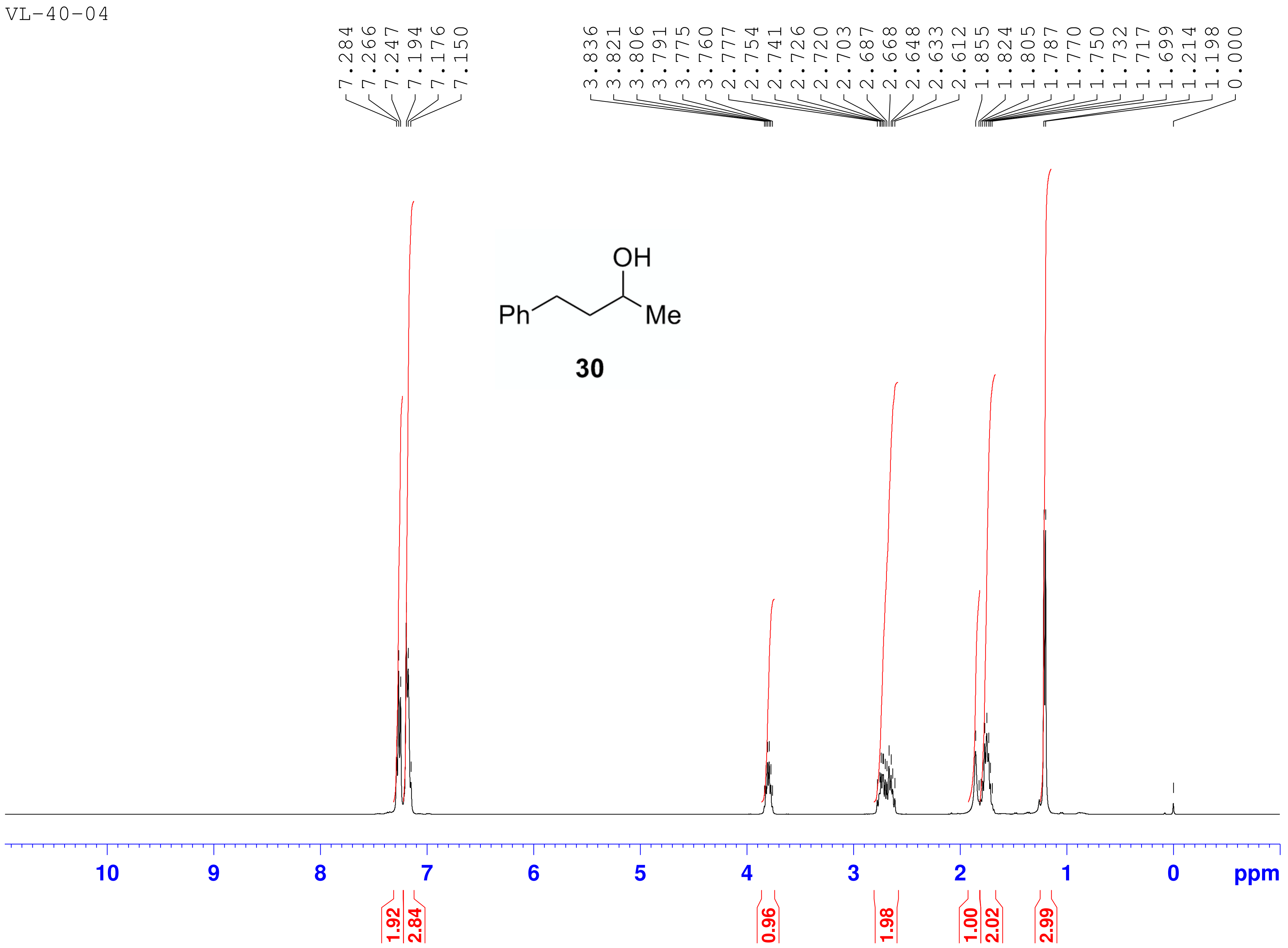
VL-40-04
7.284
7.266
7.247
7.194
7.176
7.150
3.836
3.821
3.806
3.791
3.775
3.760
2.777
2.754
2.741
2.726
2.720
2.703
2.687
2.668
2.648
2.633
2.612
1.855
1.824
1.805
1.787
1.770
1.750
1.732
1.717
1.699
1.214
1.198
0.000
OH
Ph
Me
30
10
9
8
7
6
5
4
3
2
1
0
ppm
1.92
2.84
0.96
1.98
1.00
2.02
2.99

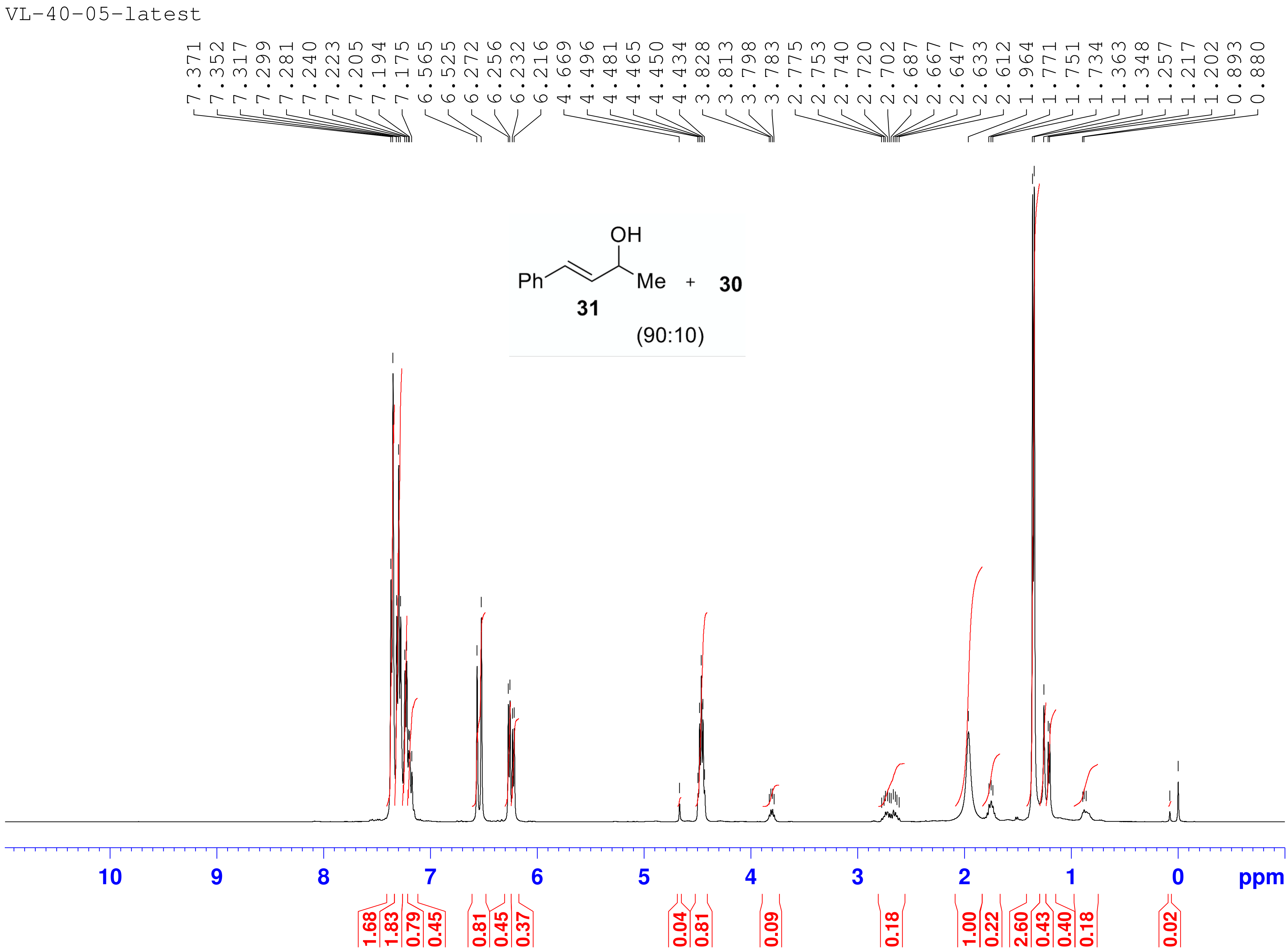
VL-40-05-latest
7.371 7.352 7.317 7.299 7.281 7.240 7.223 7.205 7.194 7.175 6.565 6.525 6.272 6.256 6.232 6.216 4.669 4.496 4.481 4.465 4.450 4.434 3.828 3.813 3.798 3.783 2.775 2.753 2.740 2.720 2.702 2.687 2.667 2.647 2.633 2.612 1.964 1.771 1.751 1.734 1.363 1.348 1.257 1.217 1.202 0.893 0.880
OH
Ph
Me
31
+ 30
(90:10)
10 9 8 7 6 5 4 3 2 1 0 ppm
1.68 1.83 0.79 0.45 0.81 0.45 0.37 0.04 0.81 0.09 0.18 1.00 0.22 2.60 0.43 0.40 0.18 0.02

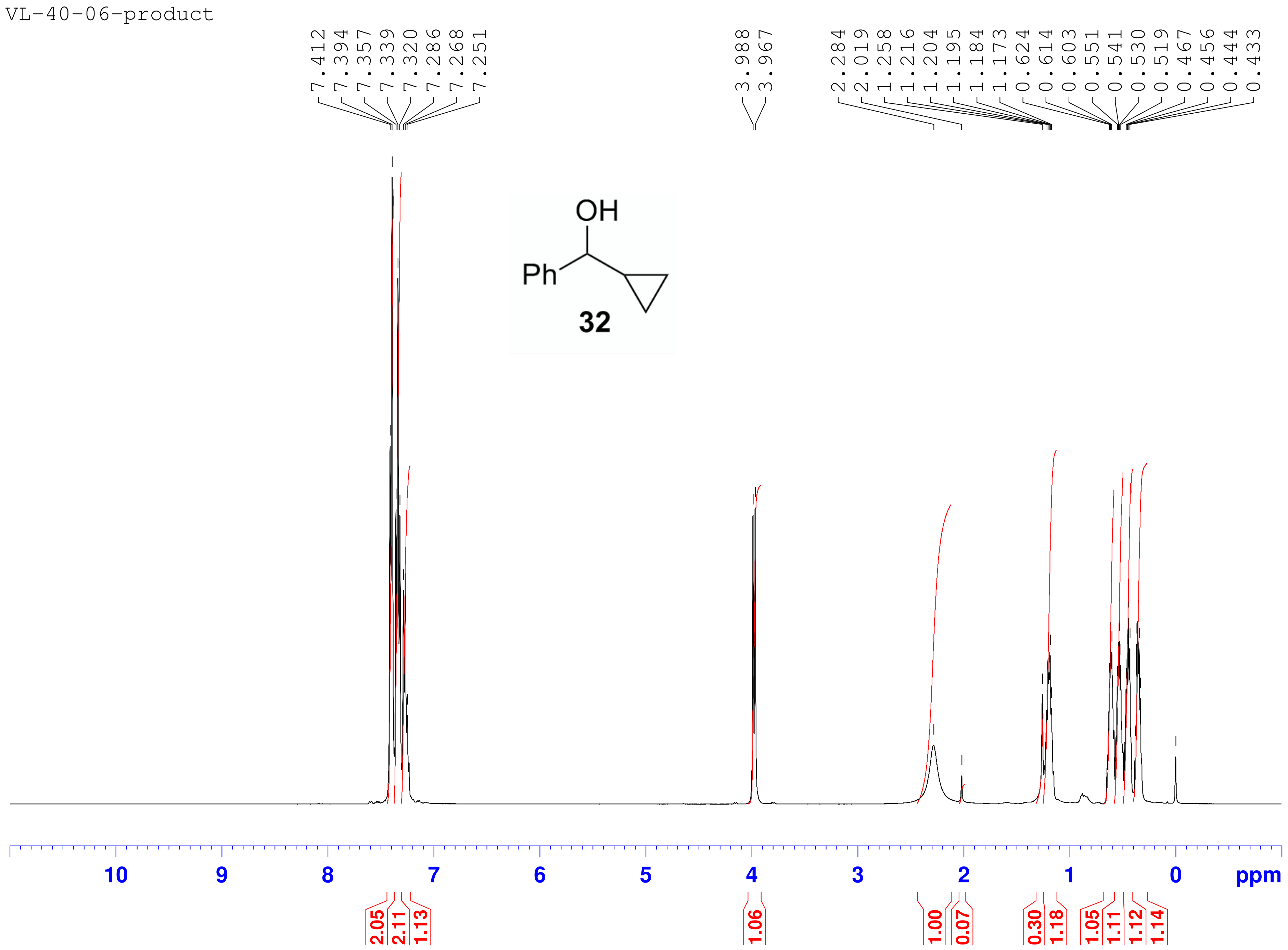
VL-40-06-product
7.412
7.394
7.357
7.339
7.320
7.286
7.268
7.251
3.988
3.967
2.284
2.019
1.258
1.216
1.204
1.195
1.184
1.173
0.624
0.614
0.603
0.551
0.541
0.530
0.519
0.467
0.456
0.444
0.433
OH
Ph
32
10
9
8
7
6
5
4
3
2
1
0
ppm
2.05
2.11
1.13
1.06
1.00
0.07
0.30
1.18
1.05
1.11
1.12
1.14

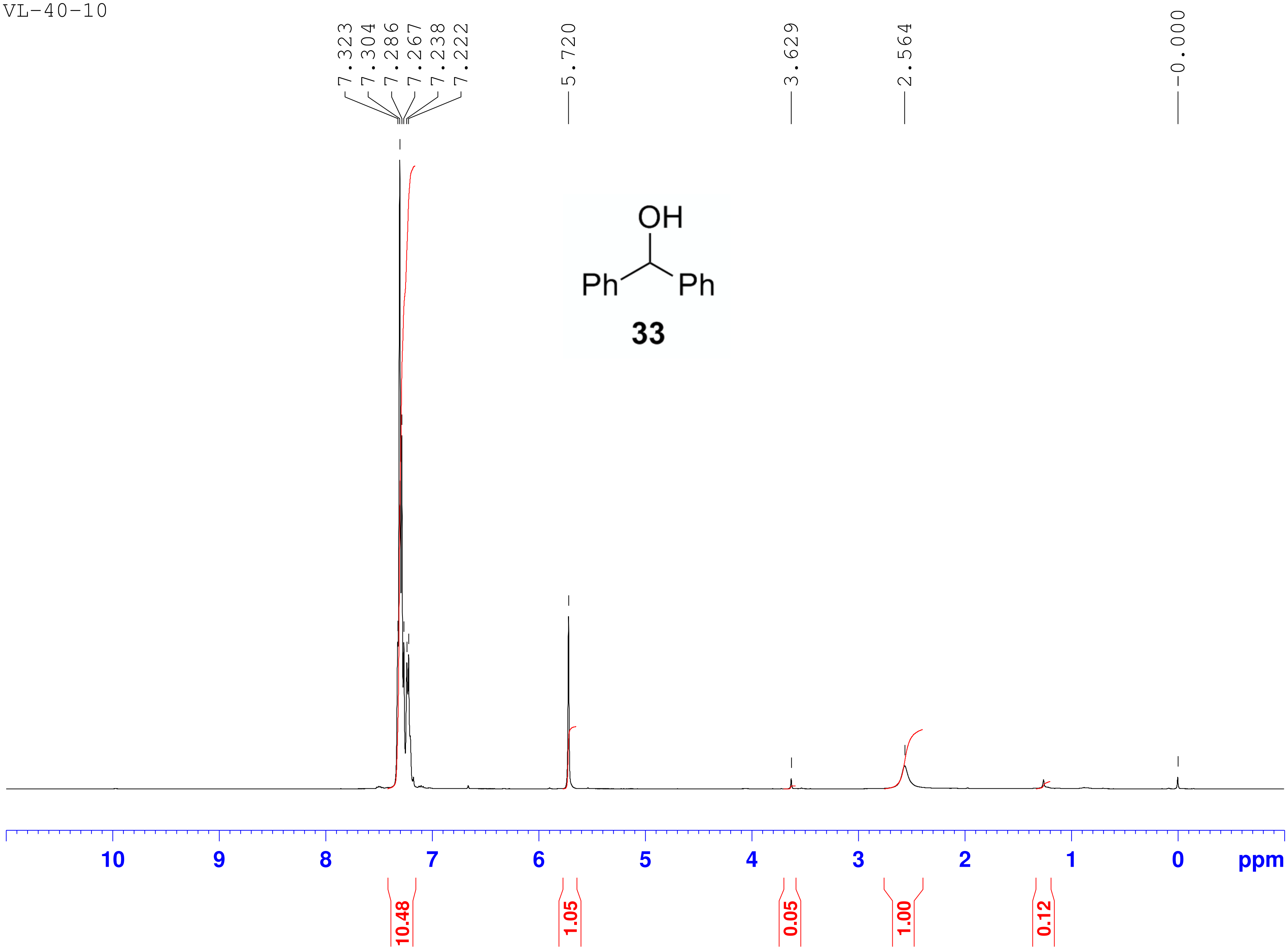
VL-40-10
7.323
7.304
7.286
7.267
7.238
7.222
5.720
3.629
2.564
-0.000
OH
Ph
Ph
33
10
9
8
7
6
5
4
3
2
1
0
ppm
10.48
1.05
0.05
1.00
0.12

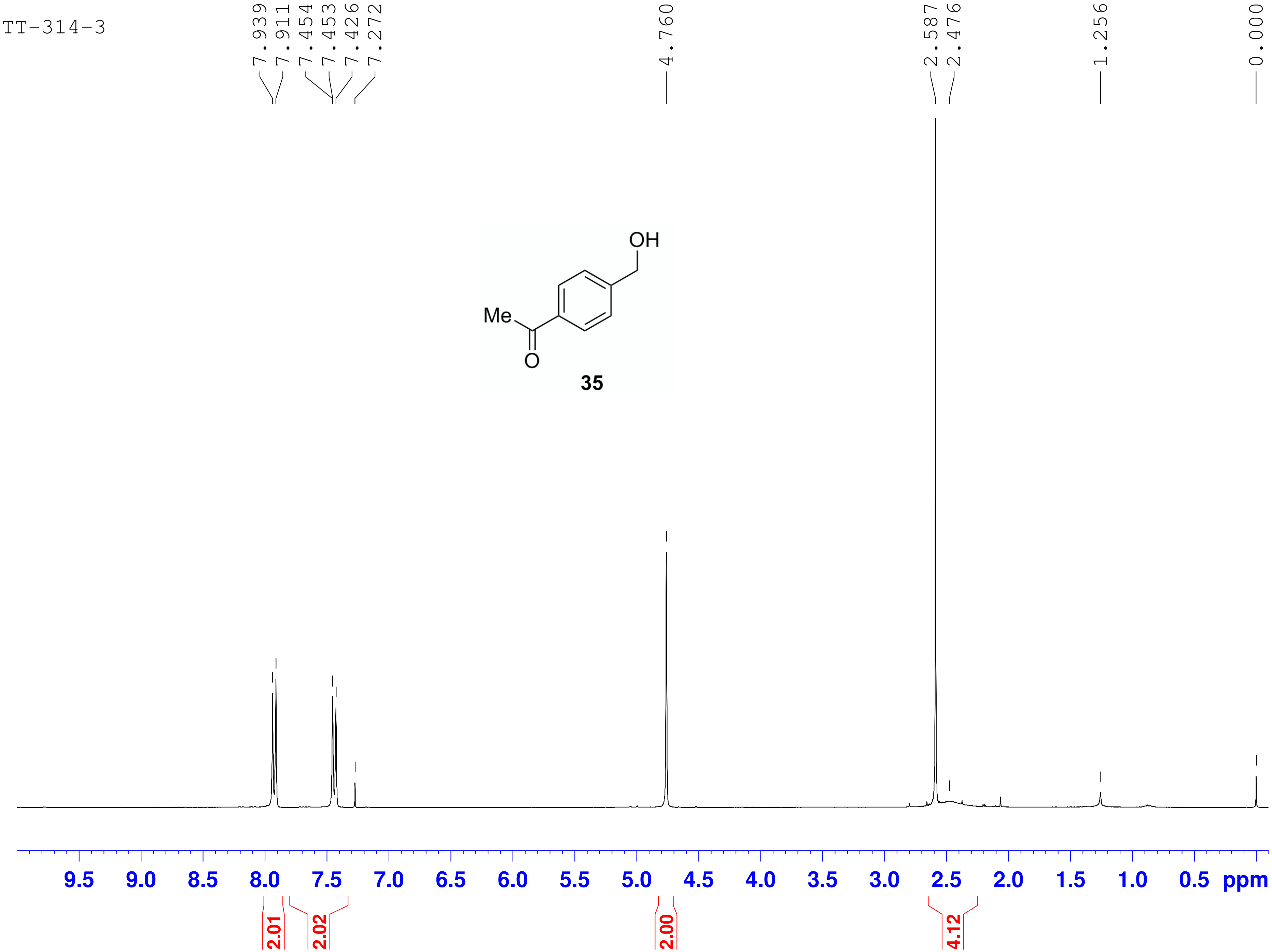
TT-314-3
7.939
7.911
7.454
7.453
7.426
7.272
4.760
2.587
2.476
1.256
0.000
OH
Me
O
35
9.5
9.0
8.5
8.0
7.5
7.0
6.5
6.0
5.5
5.0
4.5
4.0
3.5
3.0
2.5
2.0
1.5
1.0
0.5
ppm
2.01
2.02
2.00
4.12
